# Regulation of lipid metabolism in *Spodoptera frugiperda* by the symbiotic bracovirus of the gregarious parasitoid *Cotesia ruficrus*

**DOI:** 10.1371/journal.ppat.1013605

**Published:** 2025-10-17

**Authors:** Xian Li, Jun-Long An, Wen-Qin Yang, Tong-Xian Liu, Shi-Ze Zhang

**Affiliations:** 1 State Key Laboratory of Crop Stress Biology for Arid Areas, Key Laboratory of Plant Protection Resources and Pest Management of Ministry of Education, Key Laboratory of Integrated Pest Management on Crops in Northwestern Loess Plateau of Ministry of Agriculture and Rural Affairs, College of Plant Protection, Northwest A&F University, Yangling, China; 2 Institute of Entomology, College of Agriculture, Guizhou University, Guiyang, China; Boston Children's Hospital, UNITED STATES OF AMERICA

## Abstract

Parasitoids alter host energy homeostasis to create a favorable environment for their own development. However, the mechanisms underlying this process remain largely unexplored, especially for gregarious parasitoids. *Cotesia ruficrus*, a gregarious endoparasitoid native to China, targets the invasive pest *Spodoptera frugiperda* (fall armyworm, FAW) and has been shown to effectively control FAW populations. This study investigates the role of the polydnavirus (PDV) produced by *C. ruficrus* in regulating lipid metabolism of FAW larvae. The results demonstrated that, following PDV injection for 5 days, both triglyceride concentrations and lipid droplet diameters in the fat bodies of FAW larvae significantly increased. RNA interference (RNAi) targeting the PDV gene *CrBV3–31* led to a reduction in triglyceride concentrations and lipid droplet size, along with an upregulation of the *LSD1* gene. Furthermore, silencing *CrBV3–31* decreased triglyceride levels in *C. ruficrus* pupae and lowered its eclosion rate. These findings suggest that the PDV gene *CrBV3–31* plays a crucial role in enhancing lipid accumulation in FAW larvae, thereby supporting the survival of *C. ruficrus* offspring. This study uncovers a novel mechanism by which gregarious endoparasitoids exploit symbiotic bracovirus genes to regulate host energy metabolism, increasing lipid levels to meet the developmental needs of their multiple offspring.

## Author summary

Parasitoids influence the physiological metabolism of their hosts, creating an environment conducive to their own survival. However, the mechanisms underlying this phenomenon remain largely unknown, particularly for gregarious parasitoids. In this study, we investigated the gregarious parasitoid *Cotesia ruficrus*, a key native natural enemy of the invasive pest *Spodoptera frugiperda* (fall armyworm, FAW) in China. Our results showed that *C. ruficrus* stimulates an increase in the lipid levels of FAW larvae, with the parasitic factor *C. ruficrus* bracovirus (CrBV) playing a significant role. Specifically, we identified a CrBV gene, *CrBV3–31*, which inhibits the expression of *LSD1*, leading to increased lipid accumulation in FAW larvae. Moreover, *CrBV3–31* enhances the survival of *C. ruficrus* offspring. These findings shed light on how a symbiotic bracovirus gene manipulates host lipid metabolism in a gregarious parasitoid, offering valuable insights for the development of parasitoid conservation strategies.

## Introduction

Parasitoid wasps are among the most common hymenopterans that parasitize other insects, with females laying eggs either inside or on the surface of their hosts [1]. The hatched parasitoid larvae obtain nourishment directly from the host, ultimately leading to the host’s death [2]. Consequently, parasitoids play a significant role in biological control of pests. To ensure successful parasitism and the proper development of their offspring, parasitoids release a variety of parasitic factors, including venom [3], teratocytes [4], polydnaviruses (PDVs) [5], larval saliva, and ovarian proteins [6]. These parasitic factors suppress the host’s immune response, thereby mitigating the host’s defensive attacks and benefiting the parasitoid’s offspring [7,8]. Additionally, parasitoids manipulate the host’s nutritional metabolism through these factors, providing the essential nutrients needed for their development and ensuring their survival [9].

PDVs are classified into two genera: *Bracovirus* (BV), which is symbiotic with braconid wasps, and *Ichnovirus* (IV), which is symbiotic with ichneumonid wasps [10]. As a unique class of viruses, the life cycle of PDVs differs significantly from that of other viruses. While it involves typical viral processes such as replication, assembly, maturation, and spread, these occur in two distinct hosts: the parasitoid (the primary host) and the host of the parasitoid (the secondary host) [11]. Replication and assembly of PDVs occur exclusively within the primary host, specifically in the oocysts of the ovarian calyx region in female parasitoids. In the secondary host, the virus integrates into the host genome, where its virulence genes are expressed, modulating the host’s physiology to ensure the survival of the parasitoid’s offspring [12]. The PDV genome is large and fragmented, consisting of multiple circular double-stranded DNA molecules of varying lengths, each carrying different virulence genes [13]. To date, the genomes of PDVs from 11 braconid wasp species have been sequenced, including *Chelonus inanitus* bracovirus [14], *Cotesia congregata* bracovirus [15], *Microplitis demolitor* bracovirus [16], *Glyptapanteles indiensis* bracovirus and *Glyptapanteles flavicoxis* bracovirus [17], *Cotesia plutellae* bracovirus [18], *Toxoneuron nigriceps* bracovirus [19], *Cotesia vestalis* bracovirus [20], *Cotesia sesamiae Kitale* bracovirus and *Cotesia sesamiae Mombasa* bracovirus [21], and *Diolcogaster facetosa* bracovirus (ASM283394v2, Jun 28, 2019). These studies have provided valuable insights into the PDV genome, facilitating further investigation into the functions of individual viral genes. Additionally, research has shown that PDV genes can alter the immune response of the host, including proteins such as C-terminal leucine/isoleucine-rich proteins (CLPs), C-type lectins, and bracovirus apoptosis-inducing proteins (BAPs) [8,22,23]. However, there is relatively limited research on PDV genes that influence the host’s nutritional metabolism [9].

Carbohydrates, proteins, and lipids are three primary energy sources for living organisms. Among these, lipids play a particularly vital role in the biology of insects. Lipids are essential not only for growth and development but also for functions such as flight, migration, diapause, starvation, embryonic nutrition, and sex pheromone synthesis [24,25]. While lipids are found in various insect tissues, including the midgut and ovaries, the fat body serves as the primary site for lipid storage [26]. Approximately 70% of lipids are stored in the cytoplasm of fat cells within the fat body, where they are known as “lipid droplets”, and over 90% of these lipids consist of triglycerides [27,28]. The lipid content in insects is influenced by several factors, including developmental stage, diet, sex, and temperature [29,30]. Studies have shown that parasitization by parasitoids can alter the lipid content of their host insects. For instance, *Phormia regina* parasitized by *Nasonia vitripennis* results in a continuous reduction in lipid content in the host’s fat body [31]. Similarly, the ectoparasitic wasp *Bracon nigricans* induces a decrease in lipid levels in the hemolymph of *Spodoptera littoralis* [32].

The fall armyworm (FAW), *Spodoptera frugiperda* (Lepidoptera: Noctuidae), is a highly destructive pest characterized by its broad host range, strong reproductive capacity, migratory behavior, and significant crop damage [33]. Since its invasion of Yunnan, China, in 2019, FAW has spread to 27 provinces, posing a serious threat to the country’s corn production and resulting in substantial economic losses [34]. Current control measures primarily rely on chemical pesticides, which have led to environmental pollution and the development of pesticide resistance [35,36]. Consequently, there is an urgent need to develop alternative, sustainable control strategies, such as biological control technologies, which have shown promise. Field surveys in China have identified the indigenous parasitoid *Cotesia ruficrus* (Hymenoptera: Braconidae) as a natural enemy of FAW [37]. Preliminary studies suggest that *C. ruficrus* is a gregarious endoparasitoid, parasitizing FAW larvae from the first to the third instar [38]. Parasitoid wasps have evolved distinct life history strategies to optimize host utilization. These strategies, particularly in terms of the number of larvae that develop within a single host, have diverged into two primary forms: solitary and gregarious parasitism. In solitary parasitism, only one parasitoid larva develops and feeds on the host, while in gregarious parasitism, multiple larvae simultaneously develop within the host, sharing its resources [39]. Parasitization by *C. ruficrus* significantly reduces feeding activity in FAW larvae, ultimately causing their death before pupation [40,41]. Therefore, *C. ruficrus* represents a promising local candidate for the biological control of the invasive FAW.

Our research has shown that *C. ruficrus* parasitization increases the lipid content in FAW larvae, although the underlying mechanism remains unclear. Here, we report a crucial role of a *C. ruficrus* polydnavirus, named *C. ruficrus* bracovirus (CrBV), which increases host lipid levels. We further found that one CrBV gene, *CrBV3–31*, inhibited the expression of *LSD1*, which in turn increased host lipid levels. Furthermore, *CrBV3–31* benefits the survival of *C. ruficrus* offspring. This study uncovers a novel mechanism by which gregarious endoparasitoids exploit symbiotic bracovirus genes to regulate host energy metabolism, increasing lipid levels to meet the developmental needs of their multiple offspring.

## Results

### CrBV promotes lipid accumulation in FAW larvae

**Fig 1:**
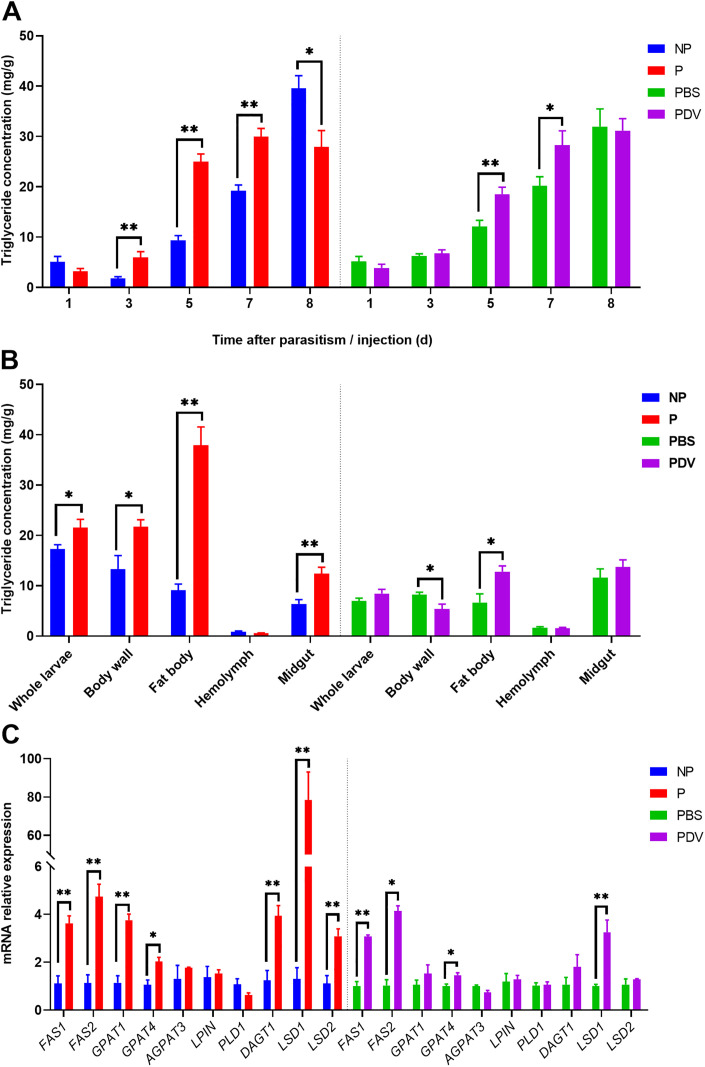
Effect of parasitism and PDV injection on triglyceride concentration and lipid metabolism-related gene expression in FAW larvae. A. Triglyceride concentration in the body wall (including fat body) of FAW larvae. B. Triglyceride concentration in various tissues of FAW larvae. C. Expression of lipid metabolism-related genes in FAW larvae. NP and P represent non-parasitized and parasitized FAW larvae, respectively. PBS and PDV refer to PBS-injected and PDV-injected FAW larvae, respectively. Data were analyzed using Student’s t-test. Values are presented as means ± SE. *, ** indicate significant differences between parasitized and non-parasitized (PBS-injected and PDV-injected) FAW larvae at **P* *< 0.05 and **P* *< 0.01, respectively.

After determining the absorbance of the sample, the triglyceride concentration in the body wall (including the fat body) of FAW larvae was calculated. It was found that the triglyceride concentration in the body wall of parasitized FAW larvae was significantly higher than that of non-parasitized FAW larvae at 3 days (*t* = 3.558, *df* = 8, *P* = 0.017), 5 days (*t* = 8.766, *df* = 8, *P* < 0.001), and 7 days (*t* = 5.401, *df* = 8, *P* = 0.001) post-parasitism. However, by day 8, the triglyceride concentration was significantly reduced (*t* = -2.832, *df* = 8, *P* = 0.022). Similarly, the triglyceride concentration in the body wall of PDV-injected FAW larvae was significantly higher than that of PBS-injected FAW larvae on days 5 (*t* = 3.475, *df* = 8, *P* = 0.008) and 7 (*t* = 2.432, *df* = 8, *P* = 0.041) (Fig 1A).

Five days post-parasitism, triglyceride concentrations were significantly higher in the whole body (*t* = -2.302, *df* = 8, *P* = 0.049), body wall (*t* = -2.822, *df* = 8, *P* = 0.022), fat body (*t* = -7.458, *df* = 8, *P* < 0.001), and midgut (*t* = -4.009, *df* = 8, *P* = 0.004) of parasitized FAW larvae compared to non-parasitized larvae. Similarly, five days after PDV injection, triglyceride concentrations in the fat body of PDV-injected larvae were significantly higher than in PBS-injected larvae (*t* = -2.972, *df* = 8, *P* = 0.018), whereas triglyceride concentrations in the body wall were significantly lower (*t* = 2.746, *df* = 8, *P* = 0.035) (Fig 1B).

Additionally, after 5 days of parasitism, the expression of lipid metabolism-related genes in FAW larvae was significantly altered. The expression of genes including *FAS1* (*t* = -5.716, *df* = 6, *P* = 0.001), *FAS2* (*t* = -5.814, *df* = 6, *P* = 0.001), *GPAT1* (*t* = -6.766, *df* = 6, *P* = 0.001), *GPAT4* (*t* = -3.566, *df* = 6, *P* = 0.012), *DGAT1* (*t* = -4.474, *df* = 6, *P* = 0.004), *LSD1* (*t* = -5.269, *df* = 6, *P* = 0.002), and *LSD2* (*t* = -4.410, *df* = 6, *P* = 0.005) was significantly upregulated in parasitized larvae compared to non-parasitized controls. Similarly, after 5 days of PDV injection, the expression of lipid metabolism genes was also upregulated in PDV-injected FAW larvae compared to PBS-injected larvae. Specifically, *FAS1* (*t* = -10.727, *df* = 6, *P* = 0.009), *FAS2* (*t* = -9.626, *df* = 6, *P* = 0.011), *GPAT4* (*t* = -3.325, *df* = 6, *P* = 0.021), and *LSD1* (*t* = -15.879, *df* = 6, *P* = 0.004) exhibited significant upregulation (Fig 1C).

**Fig 2:**
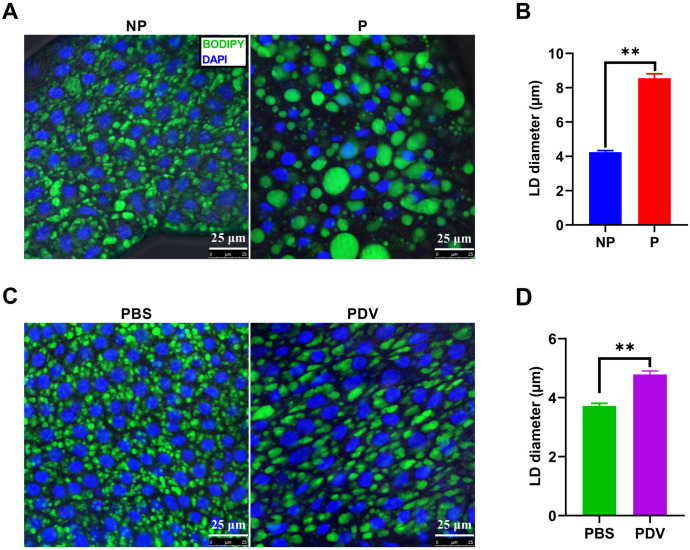
Effect of parasitism and PDV injection on lipid droplet size in the fat body of FAW larvae. A. Lipid droplet staining in the fat body of non-parasitized and parasitized FAW larvae. B. Diameter of lipid droplets in the fat body of non-parasitized and parasitized FAW larvae. C. Lipid droplet staining in the fat body of FAW larvae injected with PBS or PDV. D. Diameter of lipid droplets in the fat body of FAW larvae injected with PBS or PDV. NP and P represent non-parasitized and parasitized FAW larvae, respectively. PBS and PDV refer to PBS-injected and PDV-injected FAW larvae, respectively. Data were analyzed using Mann-Whitney U-test. Values are presented as means ± SE. ** indicates significant differences between non-parasitized and parasitized (PBS-injected and PDV-injected) FAW larvae at *P* < 0.01.

Triglycerides are stored in lipid droplets within the fat body cells of FAW larvae. To investigate changes in lipid droplet size, we stained the lipid droplets with BODIPY and measured their size. The results revealed that the lipid droplets in FAW larvae parasitized for 5 days were significantly larger than those in non-parasitized FAW larvae (*Z* = -15.328, *P* < 0.001) (Fig 2A and 2B). Similarly, the lipid droplets in FAW larvae injected with PDV for 5 days were significantly larger than those in PBS-injected larvae (*Z* = -7.725, *P* < 0.001) (Fig 2C and 2D).

### Genomic analysis of CrBV

We observed the mature CrBV particles using transmission electron microscopy, revealing cylindrical capsids with a consistent width of approximately 40 nm, but varying lengths ranging from 100 to 200 nm (S1A Fig). The CrBV genome spans 503,647 bp, consisting of 27 contigs, with sizes ranging from 3,473 bp to 46,703 bp. Notably, CrBV-25 represents a partial sequence (S1 and S2 Tables).

Through gene prediction, we identified a total of 483 complete coding sequences (CDS) and detected five tRNA genes as non-coding RNAs. Additionally, our analysis revealed 25 genomic islands (S3 Table). A comprehensive overview of the genome, including sequencing depth, GC content distribution, GC skew, and genome structure annotation, is presented in a circular genome plot (S1B Fig). We identified 10 gene families within the CrBV genome, with the protein tyrosine phosphatase (PTP) family being the most abundant, containing 27 genes. The second largest gene family, encoding the BEN domain, consists of nine genes (S4 Table and S1C Fig).

Comparative analysis with other bracoviruses revealed that most bracoviruses possess 20–30 circular double-stranded DNA molecules and have a GC content of 30%-40% (S5 Table). Phylogenetic analysis of the Lectin C-type (LCT) domain of CrBV, along with those from seven other species, showed that CrBV’s LCT domain is most closely related to that of *Cotesia sesamiae* Kitale bracovirus (S2 Fig). Similarly, a phylogenetic tree of the four Ankyrin (ANK) proteins of CrBV, compared with those from 48 other species, demonstrated that CrBV’s ANKs cluster with those from *Cotesia* species (S3 Fig).

### Expression of the CrBV gene in FAW larvae

**Fig 3:**
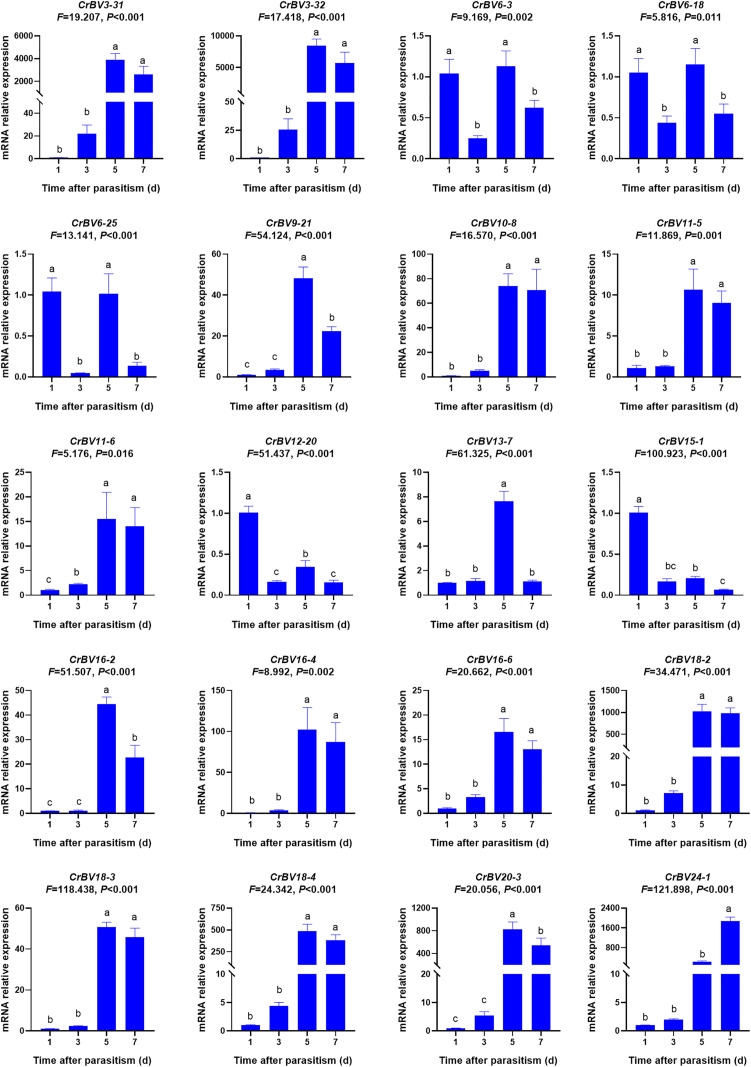
Expression of 20 CrBV genes in FAW at different time points following parasitism by *Cotesia ruficrus.* Data were analyzed using one-way ANOVA (Duncan’s multiple range test). Values are expressed as means ± SE. Different letters indicate significant differences in gene expression at different time points (**P* *< 0.05).

The transcriptomes of three FAW larvae samples parasitized by *C. ruficrus* were mapped to the CrBV genome, revealing the co-expression of 221 CrBV genes across the three samples (S4A Fig). Based on Log_2_ (FPKM) values, the top 20 highly expressed CrBV genes were selected for further analysis of their expression in FAW larvae (S4B Fig). Significant differences in gene expression were observed at various parasitism stages. After 1 day of parasitism, five genes exhibited high expression levels (*CrBV6–3*, *CrBV6–18*, *CrBV6–25*, *CrBV12–20*, and *CrBV15–1*). After 5 days, 17 genes were highly expressed (*CrBV3–31*, *CrBV3–32*, *CrBV6–3*, *CrBV6–18*, *CrBV6–25*, *CrBV9–21*, *CrBV10–8*, *CrBV11–5*, *CrBV11–6*, *CrBV13–7*, *CrBV16–2*, *CrBV16–4*, *CrBV16–6*, *CrBV18–2*, *CrBV18–3*, *CrBV18–4*, and *CrBV20–3*), and after 7 days, 11 genes showed elevated expression (*CrBV3–31*, *CrBV3–32*, *CrBV10–8*, *CrBV11–5*, *CrBV11–6*, *CrBV16–4*, *CrBV16–6*, *CrBV18–2*, *CrBV18–3*, *CrBV18–4*, and *CrBV24–1*) (Fig 3).

**Fig 4:**
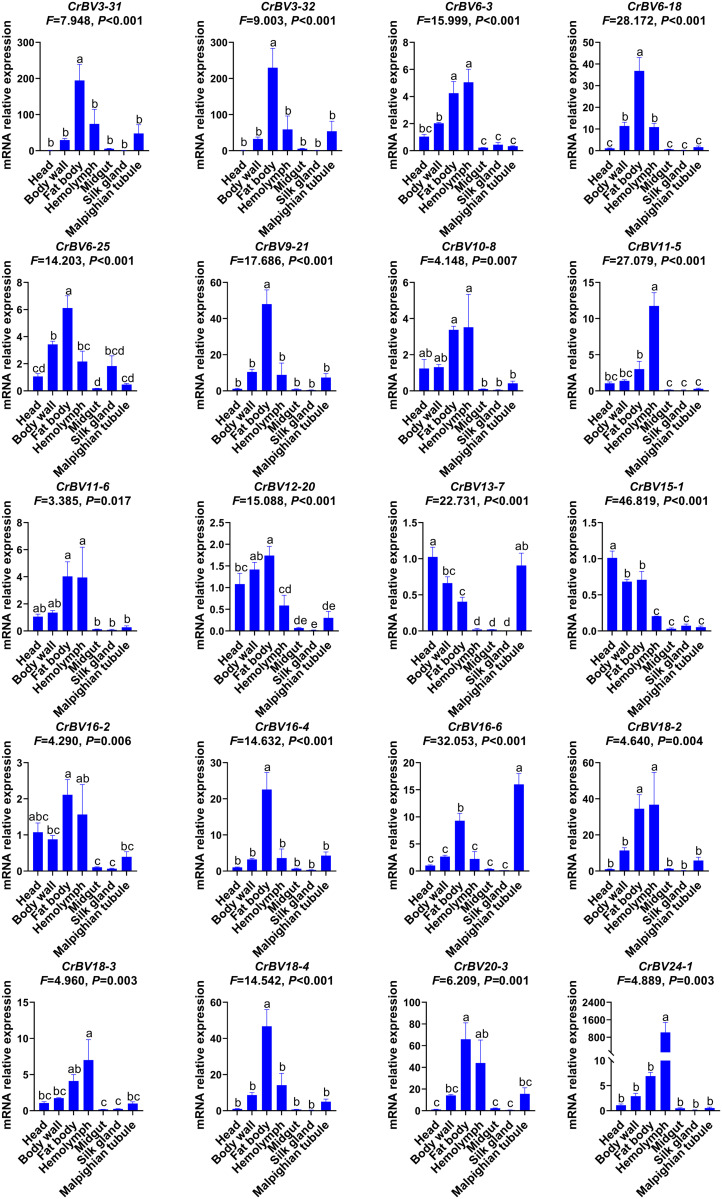
Expression of 20 CrBV genes in different tissues of FAW following parasitism by *Cotesia ruficrus.* Data were analyzed using one-way ANOVA (Duncan’s multiple range test). Values are presented as means ± SE. Different letters indicate significant differences in gene expression across tissues (*P* < 0.05).

Additionally, expression of the 20 CrBV genes varied across different FAW larval tissues following 5 days of parasitism. High expression was most prominent in the fat body and blood lymph, with 14 genes highly expressed in the fat body (*CrBV3–31*, *CrBV3–32*, *CrBV6–3*, *CrBV6–18*, *CrBV6–25*, *CrBV9–21*, *CrBV10–8*, *CrBV11–6*, *CrBV12–20*, *CrBV16–2*, *CrBV16–4*, *CrBV18–2*, *CrBV18–4*, and *CrBV20–3*) and seven genes highly expressed in the blood lymph (*CrBV6–3*, *CrBV10–8*, *CrBV11–5*, *CrBV11–6*, *CrBV18–2*, *CrBV18–3*, and *CrBV24–1*). Two genes were highly expressed in the head (*CrBV13–7* and *CrBV15–1*), while two genes were highly expressed in the Malpighian tubules (*CrBV13–7* and *CrBV16–6*) (Fig 4).

### *CrBV3–31* contributes to the accumulation of lipids in FAW larvae

**Fig 5:**
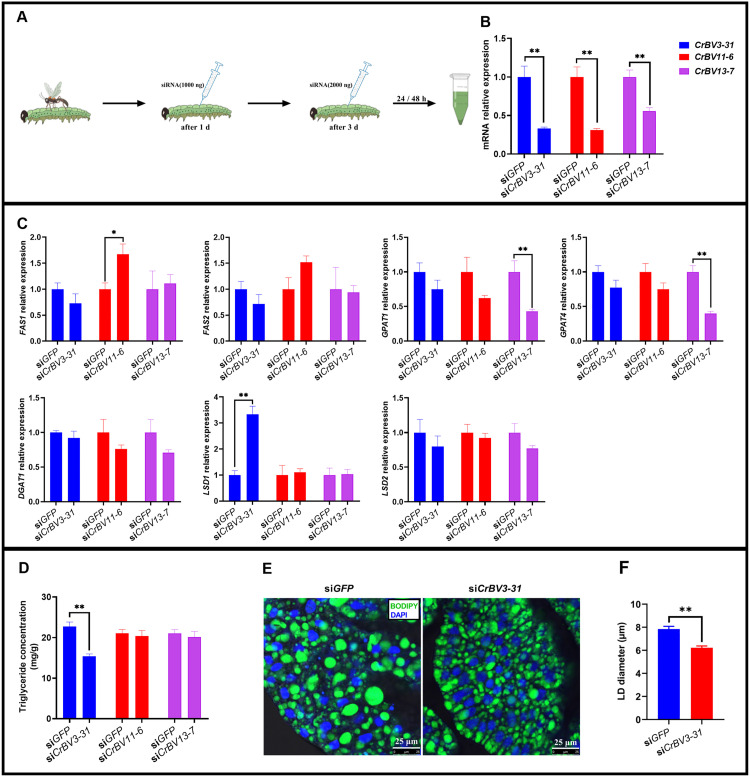
Effect of *CrBV3-31* interference on lipid metabolism in FAW larvae. A. SiRNA injection method. B. Expression of *CrBV3-31* in FAW larvae. Data were analyzed using Student’s t-test. C. Expression levels of *FAS1*, *FAS2*, *GPAT1*, *GPAT4*, *DGAT1*, *LSD1*, and *LSD2* in FAW larvae. Data were analyzed using Student’s t-test. D. Triglyceride concentration in the fat body of FAW larvae. Data were analyzed using Student’s t-test. E. Lipid droplet staining of the fat body of FAW larvae. F. Diameter of lipid droplets in the fat body of FAW larvae. Data were analyzed using Mann-Whitney U-test. si*GFP* and si*CrBV3-31* refer to FAW larvae injected with si*GFP* and si*CrBV3-31*, respectively. Values are presented as means ± SE. *, ** indicate statistically significant differences between si*GFP*- and si*CrBV3-31*-injected larvae at **P* *< 0.05 and **P* *< 0.01, respectively.

To investigate the effects of *CrBV3–31*, *CrBV11–6* and *CrBV13–7* on lipid metabolism in parasitized FAW larvae, we employed RNA interference (RNAi) to silence *CrBV3–31*, *CrBV11–6* and *CrBV13–7*. The results demonstrated that the gene expression of *CrBV3–31* (*t* = 4.706, *df* = 10, *P* = 0.005), *CrBV11–6* (*t* = 4.529, *df* = 10, *P* = 0.001), and *CrBV13–7* (*t* = 4.292, *df* = 10, *P* = 0.004) was significantly downregulated at 48 hours post-interference (Fig 5A and 5B).

SiRNA targeting *CrBV3–31* was injected into parasitized FAW larvae, resulting in a significant reduction in triglyceride concentrations in the fat body compared to the control group injected with si*GFP*, 48 hours after treatment (*t* = 5.736, *df* = 8, *P* < 0.001) (Fig 5D). Additionally, the diameter of lipid droplets in the fat body was significantly smaller in the experimental group than in the control (*Z* = -6.187, *P* < 0.001) (Fig 5E and 5F). However, when SiRNAs targeting *CrBV11–6* (*t* = 0.391, *df* = 8, *P* = 0.706) and *CrBV13–7* (*t* = 0.528, *df* = 8, *P* = 0.612) were injected into parasitized FAW larvae, no significant change in triglyceride concentration was observed in the fat body (Fig 5D). Furthermore, the expression of the FAW gene *FAS1* was significantly upregulated following *CrBV11–6* silencing (*t* = -3.068, *df* = 10, *P* = 0.012). The expression of the FAW gene *GPAT1* (*t* = 3.529, *df* = 10, *P* = 0.005) and *GPAT4* (*t* = 6.084, *df* = 10, *P* = 0.001) was significantly downregulated following *CrBV13–7* silencing, while the expression of the FAW gene *LSD1* was significantly upregulated following *CrBV3–31* silencing (*t* = -6.686, *df* = 10, *P* < 0.001) (Fig 5C). A phylogenetic tree of the LSD1 protein from FAW and 56 other species revealed that FAW’s LSD1 clustered with that of *Spodoptera litura*, suggesting a closer genetic relationship between the two species (S5 Fig).

Additionally, we investigated the effects of silencing *CrBV3–31*, *CrBV11–6*, and *CrBV13–7* on the expression of other CrBV genes. The results showed that silencing *CrBV3–31* led to a downregulation of *CrBV3–32*, *CrBV18–2*, and *CrBV18–4* (S6 Fig). Silencing *CrBV11–6* resulted in the downregulation of *CrBV9–21*, *CrBV11–5*, *CrBV15–1* and *CrBV18–3*, while *CrBV10–8* expression was upregulated (S7 Fig). Silencing *CrBV13–7* caused a downregulation of *CrBV21–4*, while *CrBV3–31*, *CrBV3–32*, *CrBV6–3*, *CrBV6–18*, *CrBV6–25*, *CrBV10–8*, *CrBV11–5*, and *CrBV16–2* were upregulated (S8 Fig).

### *CrBV3–31* is beneficial for improving the performance of *C. ruficrus* offspring

**Fig 6:**
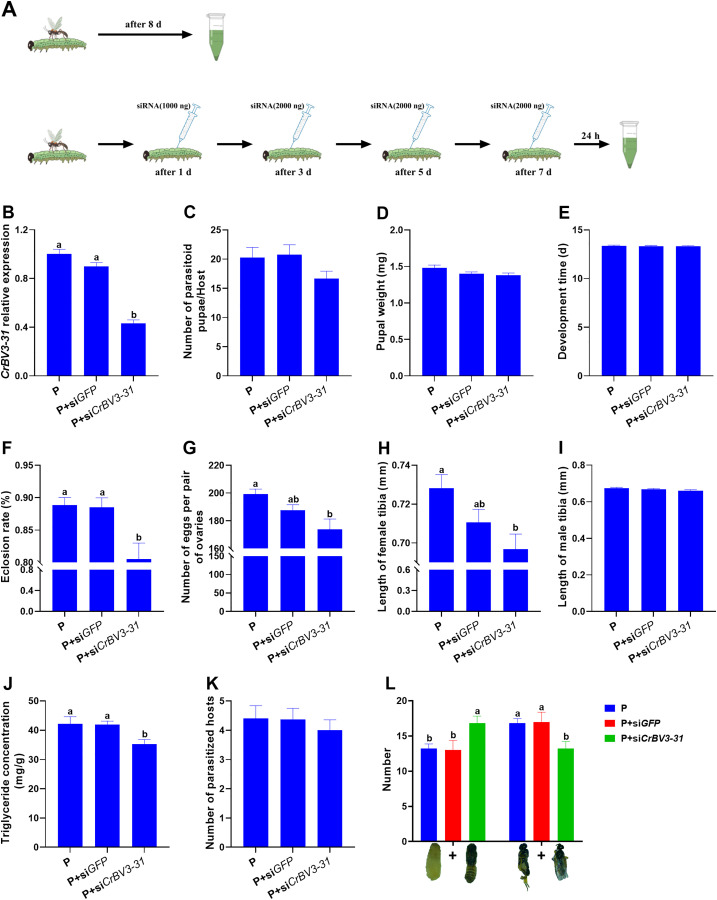
Effect of *CrBV3-31* interference on the performance of *Cotesia ruficrus* offspring. A. SiRNA injection method. B. Expression of *CrBV3-31* in FAW larvae. Data were analyzed by one-way ANOVA (Duncan’s test). C. Number of parasitoid pupae per host. Data were analyzed by Kruskal-Wallis H-test (Duncan’s test). D. Pupal weight of *C. ruficrus*. Data were analyzed by one-way ANOVA (Duncan’s test). E. Development time of *C. ruficrus*. Data were analyzed by Kruskal-Wallis H-test (Duncan’s test). F. Eclosion rate of *C. ruficrus* pupae. Data were analyzed by Kruskal-Wallis H-test (Duncan’s test). G. Number of eggs per pair of ovaries of *C. ruficrus*. Data were analyzed by Kruskal-Wallis H-test (Duncan’s test). H. Length of the hind tibia of female *C. ruficrus* adults. Data were analyzed by one-way ANOVA (Duncan’s test). I. Length of the hind tibia of male *C. ruficrus* adults. Data were analyzed by one-way ANOVA (Duncan’s test). J. Triglyceride concentration in *C. ruficrus* pupae. Data were analyzed by one-way ANOVA (Duncan’s test). K. Number of parasitized FAW larvae. Data were analyzed by one-way ANOVA (Duncan’s test). L. Number of non-eclosive *C. ruficrus* in different developmental states. Data were analyzed by one-way ANOVA (Duncan’s test). P, P + si*GFP*, and P + si*CrBV3-31* represent non-injected, si*GFP*-injected, and si*CrBV3-31*-injected FAW larvae, respectively. Values are presented means ± SE. Different letters indicate significant differences at *P* < 0.05.

To investigate the effects of *CrBV3–31* gene on the offspring of *C. ruficrus* parasitizing FAW larvae, we employed RNAi to silence *CrBV3–31*. The results showed that after four consecutive interferences with *CrBV3–31* gene, the expression level of the gene was significantly downregulated 24 hours post-interference (*F*_2,12_ = 80.585, *P* < 0.001) (Fig 6A and 6B).

After parasitized FAW larvae were injected with si*CrBV3–31*, the eclosion rate of *C. ruficrus* pupae (*χ*^2^ = 6.897, *P *= 0.032) and the triglyceride concentration in one-day-old *C. ruficrus* pupae (*F*_2,12_* *= 4.496, *P *= 0.035) were significantly lower compared to those of si*GFP*-injected and non-injected parasitized FAW larvae (Fig 6F and 6J). Additionally, the number of eggs per pair of ovaries (*χ*^2^ = 7.910, *P *= 0.019) and the hind tibia length of female *C. ruficrus* adults (*F*_2,87_* *= 4.910, *P *= 0.010) were significantly smaller in the si*CrBV3–31-*injected than in the non-injected parasitized FAW (Fig 6G and 6H). However, no significant changes were observed in the number of parasitoid pupae per host (Fig 6C: *χ*^2^ = 3.859, *P *= 0.145), pupal weight (Fig 6D: *F*_2,27_ = 2.779, *P* = 0.080), developmental time (Fig 6E: *χ*^2^ = 0.156, *P *= 0.925) of *C. ruficrus*, hind tibia length of male *C. ruficrus* adults (Fig 6I: *F*_2,87_* *= 1.550, *P *= 0.218), or number of parasitized FAW larvae (Fig 6K: *F*_2,39_ = 0.231, *P* = 0.795).

Further dissection of parasitoid cocoons that did not successfully emerge revealed four developmental categories: a) white pupae; b) black pupae; c) malformed adults; and d) normal but deceased adults (S9A Fig). No significant differences were observed in the number of parasitoids in these four developmental states across the three treatments (S9B Fig). However, when the developmental status was grouped into two categories–pupae (a + b) and adults (c + d)–the number of parasitoids in the pupal stage was significantly higher in the si*CrBV3–31*-injected FAW larvae compared to the si*GFP*-injected and non-injected control groups (*F*_2,12_* *= 4.059, *P *= 0.045), while the number of parasitoids in the adult stage was significantly lower (*F*_2,12_* *= 4.059, *P *= 0.045) (Fig 6L).

## Discussion

The parasitism of host insects by parasitoids induces a range of physiological changes, including alterations in immunity, development, and nutrient metabolism. Parasitic factors such as PDV, venom, and teratocytes play significant roles in this process [42]. Our research demonstrated that the PDV from *C. ruficrus*, CrBV, influences the lipid accumulation patterns in the host larvae of FAW. Notably, the CrBV gene *CrBV3–31* is involved in regulating lipid metabolism in FAW larvae and also supports the development of *C. ruficrus* offspring.

Lipids are essential energy substrates that play a critical role throughout an organism’s life cycle [24]. Previous studies have shown that parasitic wasps can alter the lipid metabolism of their host insects [9,43]. Our findings are consistent with this, showing that the parasitoid *C. ruficrus* regulates lipid metabolism in FAW larvae. Specifically, when *C. ruficrus* parasitizes third instar FAW larvae, triglyceride concentrations significantly increase from 3 to 7 days post-parasitism, with the highest concentration observed in the fat body 5 days after parasitism. Additionally, lipid droplets in the fat body become larger. Similarly, after parasitism by *Chelonus inanitus*, fat accumulation is observed in *S. littoralis* [44]. Other studies have also reported lipid accumulation in host insects, such as *Aphis gossypii* parasitized by *Lysiphlebia japonica* and *Drosophila melanogaster* parasitized by *Leptopilina boulardi* [45,46], which aligns with our observations. However, certain parasitoids have the opposite effect, as seen in *Plutella xylostella*, where fat content decreases after parasitism by *Cotesia vestalis* [9]. We speculate that this discrepancy arises because *C. ruficrus* is a gregarious parasitoid, requiring more nutrients from the host to support the development of its larvae, whereas *C. vestalis* is a solitary parasitoid that does not rely as heavily on host nutrition. Additionally, the accumulation of nutrients in the host may enhance its immunity, which could hinder the development of *C. vestalis* larvae [26].

To further investigate the underlying mechanisms, we examined the expression of genes associated with lipid metabolism. Our results showed that the gene expression patterns corresponded with changes in triglyceride concentration and lipid droplet size. Specifically, the expression of genes involved in fatty acid synthesis, such as *FAS1* and *FAS2*, and triglyceride synthesis, including *GPAT1*, *GPAT4*, and *DGAT1*, increased post-parasitism. Additionally, genes encoding surface proteins of lipid droplets, such as *LSD1* and *LSD2*, were upregulated. *LSD1* is known to promote fat decomposition through the adipokine hormone (AKH) signaling pathway or by recruiting lipases to lipid droplet surfaces, while *LSD2* inhibits fat breakdown, thereby facilitating lipid accumulation [47,48]. The increase in *LSD1* expression observed in our study suggests that lipid degradation occurs to provide resources for the parasitoid larvae. In conclusion, our study demonstrates that parasitism by *C. ruficrus* significantly alters the lipid metabolism of FAW larvae, promoting lipid accumulation in the fat body. These changes are mediated through the regulation of key genes involved in lipid metabolism, with implications for both host physiology and parasitoid development.

Parasitoids induce changes in the lipid metabolism of their hosts, with various parasitic factors playing crucial roles in this process. Among these, PDVs are significant in Braconidae, influencing host metabolism [49]. For instance, the PDV of *C. inanitus* has been shown to promote fat accumulation in *S. littoralis* [44], while injection of the PDV from *C. vestalis* leads to a reduction in triglyceride concentration in *P. xylostella* [9]. In our study, we injected the PDV of *C. ruficrus* into FAW larvae and observed a significant increase in triglyceride concentrations 5–7 days post-injection. Five days after injection, triglyceride levels in the fat body were elevated, and lipid droplets within the fat body increased in size. These findings mirror those observed when *C. ruficrus* parasitizes FAW larvae. Additionally, the expression of genes involved in lipid metabolism, including *FAS1*, *FAS2*, *GPAT4*, and *LSD1*, was significantly upregulated, consistent with the observed increase in lipid content. Our results suggest that CrBV plays a key role in enhancing lipid accumulation in FAW larvae during specific periods following parasitism by *C. ruficrus*.

The regulation of host physiological processes by PDVs is primarily mediated by the virulence genes encoded within the PDV genome [50]. In our study, we sequenced the genome of *C. ruficrus* bracovirus (CrBV), revealing a genome size of 503.647 kb, consisting of 27 circular segments. This genomic structure is similar to that of closely related PDVs, including *C. congregata* bracovirus, *C. vestalis* bracovirus, and *C. sesamiae* Kitale bracovirus [15–21]. Like most bracoviruses, the CrBV genome encodes several conserved gene families, such as PTP, BEN domain, histone, lectin, and ribonuclease T2 [51]. In addition to these well-characterized gene families, the CrBV genome contains a large number of genes of unknown function, which warrant further investigation.

By integrating the CrBV genome with the transcriptome data from parasitized FAW larvae, we identified 221 CrBV genes that were co-expressed in the transcriptomes of three FAW larvae samples. Previous studies have shown that the expression of PDV genes in parasitized insects can begin as early as two hours post-parasitism and persists throughout the parasitic period [52–54]. While genes of ichnoviruses are generally expressed continuously during parasitism, bracovirus genes, including those of CrBV, tend to be expressed preferentially at specific times during parasitism [54]. We further analyzed the expression of the 20 most highly expressed CrBV genes at different time points following parasitism using RT-qPCR. Notably, the highest expression levels were observed 5 days after parasitism, with 17 out of the 20 genes showing peak expression at this time. Previous studies indicating that PDV genes are widely expressed across various tissues of parasitized insects but certain genes are preferentially expressed in specific host tissues or cells [55]. Hemocytes and fat bodies are typically the sites with the highest levels of PDV gene transcription [54], likely due to their accessibility to viral particles and their roles in immune defense and metabolism [56–58]. Consistent with this, our analysis of CrBV gene expression in different tissues of FAW larvae 5 days post-parasitism revealed that most of the highly expressed CrBV genes were concentrated in the fat body and hemolymph, mirroring the results of previous studies.

In the present study, we utilized RNAi to silence *CrBV3–31*, *CrBV11–6*, and *CrBV13–7* expressions. Forty-eight hours after *CrBV3–31* interference, we observed a significant upregulation of the *LSD1* gene, accompanied by a marked reduction in triglyceride concentration in the fat body of FAW larvae. Additionally, we measured the diameter of lipid droplets in the fat body of parasitized FAW larvae 48 hours after *CrBV3–31* interference. The results indicated that lipid droplets were smaller in size in the larvae subjected to *CrBV3–31* silencing compared to the controls, which correlates with the observed decrease in triglyceride concentration. Interestingly, we observed a puzzling phenomenon: following parasitism by *C. ruficrus* and PDV injection into FAW larvae, the expression of the *LSD1* gene was significantly upregulated. However, when we used si*CrBV3–31* to interfere with *CrBV3–31* expression, *LSD1* expression was not downregulated but instead further upregulated. This suggests that *CrBV3–31* may regulate *LSD1* expression, potentially through interactions with other CrBV genes. To explore this, we measured the expression of 19 other CrBV genes following *CrBV3–31* silencing and found that the expression of *CrBV3–32*, *CrBV18–2*, and *CrBV18–4* was downregulated. *LSD1* promotes fat decomposition via the adipokine hormone (AKH) signaling pathway or by recruiting lipases to lipid droplet surfaces, while *LSD2* inhibits fat breakdown, facilitating lipid accumulation. Therefore, high *LSD1* expression is correlated with smaller lipid droplets, whereas high *LSD2* expression corresponds to larger lipid droplets. In the knockdown experiments, *LSD1* expression was upregulated, while *LSD2* expression remained unchanged, leading to a reduction in lipid droplets. However, in parasitic experiments, *LSD1* expression was upregulated, which should have resulted in smaller lipid droplets, while *LSD2* was also upregulated along with triglyceride synthesis genes *GPAT1*, *GPAT4*, and *DGAT1*. This resulted in a net accumulation of lipids and larger lipid droplets. Furthermore, in the parasitic experiment, the control group consisted of healthy FAW, while the treatment group included parasitized FAW. The parasitic wasp larvae rely on the FAW host to degrade lipids for energy. Consequently, *LSD1* must be upregulated in parasitized FAW compared to healthy individuals.

After 48 hours of *CrBV11–6* interference, a significant upregulation of the *FAS1* gene was observed, but no significant change in triglyceride concentration was detected in the fat body of FAW larvae. This may be attributed to *FAS1*’s role in fatty acid synthesis, which is a precursor for lipid synthesis and operates via a pathway distinct from triglyceride synthesis [59]. Similarly, after 48 hours of silencing *CrBV13–7*, significant downregulation of the *GPAT1* and *GPAT4* genes in the glycerol-3-phosphate (G3P) (Kennedy) pathway was observed, but triglyceride concentration in the fat body of FAW larvae remained unchanged. This could be due to a compensatory effect from the monoacylglycerol acyltransferase pathway, an alternative route for triglyceride synthesis [60]. Interfering with three CrBV genes led to alterations in the expression of other CrBV genes, particularly those involved in lipid metabolism. These findings suggest that FAW lipid metabolism is influenced by the interaction of multiple CrBV genes. However, the precise mechanism remains unclear and warrants further investigation.

The nutritional status of the host is critical for the development of parasitoid larvae. After silencing *CrBV3–31*, we observed a reduction in lipid content in the host FAW larvae, which may affect parasitoid development [61]. To further investigate this hypothesis, we examined the development of *C. ruficrus* offspring following *CrBV3–31* interference in parasitized FAW larvae. We found that lipid content in the pupae of the parasitoid offspring was reduced, and the eclosion rate significantly declined. Anatomical analysis revealed that parasitoids that failed to emerge largely ceased development during the pupal stage.

**Fig 7:**
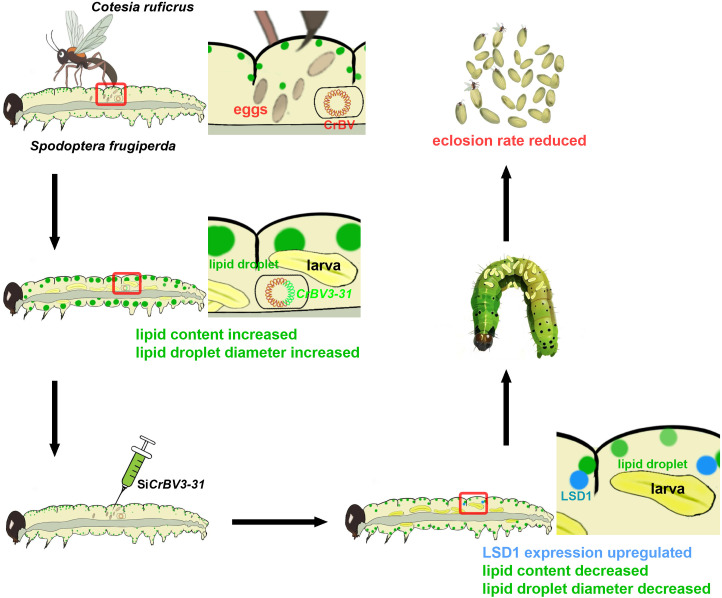
Model for manipulation of host lipid metabolism by gregarious parasitoid *Cotesia ruficrus.*

In conclusion, we have successfully sequenced the genome of *C. ruficrus* bracovirus (CrBV), providing a valuable reference for future identification of other bracovirus genomes. Our findings show that CrBV promotes lipid accumulation in FAW larvae. Additionally, *CrBV3–31* contributes to lipid accumulation prior to pupation in *C. ruficrus* and supports the successful emergence of parasitoids. These findings indicate that the gregarious parasitoid *C. ruficrus* enhances the lipid content of its host to meet the nutritional requirements of its larvae, thereby facilitating the parasitoid’s successful emergence (Fig 7). Our research also provides a foundation for future studies on how parasitoids regulate host metabolism, which could aid in the conservation and utilization of parasitoid populations.

## Materials and methods

### Insects rearing

The FAW larvae were collected from corn fields in Yangling District, Xianyang, China (34°17′N, 108°01′E) in July 2019. These larvae were reared individually on leaves of *Zea mays* L. var. Shandan 636 in the laboratory to establish a stable population. In June 2020, parasitized FAW larvae were collected from corn fields in Zhouzhi District, Xi’an, China (34°09′N, 108°13′E) and reared individually on corn leaves in the laboratory. Once parasitoid cocoons were observed, they were collected, and upon parasitoid emergence, the adults were reared using FAW larvae in the 1–3 instar stage as hosts. The parasitoid was identified as *C. ruficrus* by Professor Chen Xue-Xin of Zhejiang University. For sex differentiation of *C. ruficrus*, the method described by He et al. was followed [37].

All insect populations were maintained in an intelligent climate cabinet (Shanghai Yiheng Scientific Instrument, China) under controlled conditions: 26 ± 1°C, 60% ± 5% relative humidity, a photoperiod of 14L:10D, and supplemented with 10% honey water for nutrition. Corn was grown in enclosed net cages (60 × 60 × 60 cm, mesh size 100) under the same conditions, watered every three days, and without pesticide application. Once the corn reached the three-leaf stage, it was harvested and used to feed the FAW larvae.

### Measurement of triglyceride concentration in FAW larvae

One mated female *C. ruficrus* was placed with a 3rd instar, 1-day-old FAW larva in a test tube (diameter 1.2 cm, length 6.0 cm, with mesh lids). After parasitization was observed, the female was removed, and the FAW larvae were fed daily with corn leaves. The body wall (including the fat body) of FAW larvae were collected at 1, 3, 5, 7, and 8 days post-parasitism. At 5 days after parasitism, the larvae were dissected to obtain the body wall, fat body, hemolymph (mixed with 100 μL mineral oil (Adamas, China) to prevent melanization), and midgut. The samples were stored at -20°C for later triglyceride concentration analysis using a triglyceride assay kit (Solarbio, China). Non-parasitized FAW larvae were used as the control. Each treatment was repeated five times.

Additionally, ovaries from 100 mated *C. ruficrus* females (1-day-old) were dissected and placed in a 1.5 mL Eppendorf tube containing 100 μL of PBS (0.01 M, PH = 7.4). The ovaries were homogenized and centrifuged, and the CrBV filtrate was obtained through a 0.45 μm filter membrane. A 0.1 μL aliquot of the CrBV filtrate was injected into 1-day-old, 3rd instar FAW larvae using a Nanoinject II microinjector (Drummond Scientific, USA). The FAW larvae were fed daily with corn leaves. The body wall (including the fat body) of FAW larvae were collected at 1, 3, 5, 7, and 8 days post-injection. At 5 days after injection, the larvae were dissected to obtain the body wall, fat body, hemolymph (mixed with 100 μL mineral oil to prevent melanization), and midgut. The samples were stored at -20°C for later triglyceride concentration analysis. FAW larvae injected with 0.1 μL PBS were used as the control. Each treatment was repeated five times.

### RT-qPCR analysis of lipid metabolism genes in FAW larvae

As described in “Measurement of triglyceride concentration in FAW larvae”, parasitized FAW larvae were collected after 5 days of parasitism and stored at -20°C. Non-parasitized FAW larvae were collected as controls. Total RNA was extracted from the whole larvae using Trizol reagent (Invitrogen, USA), and reverse transcribed using a reverse transcription kit (Takara, Japan). Ten genes involved in lipid metabolism–*FAS1*, *FAS2*, *GPAT1*, *GPAT4*, *AGPAT3*, *LIPIN*, *PLD1*, *DGAT1*, *LSD1*, and *LSD2*–were selected for RT-qPCR analysis (detailed information for these genes is provided in S6 Table), with *LOC118272864* serving as the internal reference gene. Primers for the 11 gene sequences were designed using PrimerPremier 6.0 (S7 Table). Quantification was performed using the LightCycler 480 system (Roche Diagnostics, Switzerland) with the TB Green Premix Ex Taq II (Tli RNaseH Plus) reaction system (Takara, Japan). The amplification program included a pre-denaturation step at 95°C for 30 s, followed by 40 cycles of 95°C for 5 s and 60°C for 20 s. A melt curve analysis was conducted from 65 to 95°C to verify the purity of the PCR products. Each gene was analyzed in four biological replicates, with three technical replicates per sample. Data analysis was performed using the 2^−^^△△^^CT^ method.

For the injection experiments, 0.1 μL of PDV was injected into FAW larvae as described in “Measurement of triglyceride concentration in FAW larvae”. Whole larvae were collected after 5 days of injection and stored at -20°C. A control group of FAW larvae was injected with 0.1 μL of PBS. RT-qPCR analysis was performed on the same 10 lipid metabolism genes (*FAS1, FAS2, GPAT1, GPAT4, AGPAT3, LPIN, PLD1, DGAT1, LSD1, LSD2*), as outlined above, with four biological replicates and three technical replicates for each gene.

### Lipid droplet staining and size measurement in FAW larvae

As described in “Measurement of triglyceride concentration in FAW larvae”, FAW larvae were dissected in PBS after 5 days of parasitism and PDV injection to isolate the fat bodies. The fat bodies were fixed in 4% paraformaldehyde (Servicebio, China) at room temperature in the dark for 30 minutes. The samples were then washed three times with 500 μL of PBST (PBS containing 0.1% Triton and 0.05% Tween 20), each wash lasting 10 minutes. Next, the samples were incubated with 500 μL of 1 mg/mL BODIPY 493/503 (AbMole, USA) at room temperature for 30 minutes. After incubation, the samples were washed three times with 500 μL of PBST, each wash lasting 5 minutes. To stain the nuclei, 500 μL of 10 μg/mL DAPI (Solarbio, China) was added, and the samples were incubated at room temperature for 30 minutes. This was followed by three 10-minute washes with PBS. The samples were then mounted with antifade mounting medium (Solarbio, China) and observed using a Leica fluorescence confocal microscopy (Leica, Germany). Finally, the diameters of the lipid droplets were measured randomly using Adobe Photoshop 2021. Non-parasitized and PBS-injected FAW larvae served as controls. A total of 200 lipid droplets were randomly selected and analyzed for each treatment.

### Morphological analysis of CrBV

Fifty pairs of ovaries from 1-day-old mated *C. ruficrus* females were dissected and placed in a 1.5 mL EP tube containing 100 μL of PBS. The ovaries were homogenized and centrifuged at 1000 *g* for 5 minutes at 4°C. The resulting CrBV filtrate was collected through a 0.45 μm filter membrane. The filtrate was then applied to a copper mesh and negatively stained with 2% uranyl acetate dihydrate. The samples were observed and photographed using a Hitachi HT7800 transmission electron microscope (Hitachi, Japan).

### Genomic sequencing of CrBV

According to Chen et al. [19], PDV virus particles were collected and virus DNA was extracted from female wasps. Briefly, 1-day-old *C. ruficrus* females were paralyzed by placing them at 4°C. The parasitoids were then dissected in pre-chilled PBS, and 1000 pairs of ovaries were collected. The ovaries were mechanically homogenized to release ovarian fluid into the PBS. The fluid was filtered through a 0.45 μm filter to remove eggs and cellular debris. The filtered viral fluid was treated with nucleases to eliminate contamination from parasitoid genomic DNA and other exogenous nucleic acids. CrBV DNA was subsequently extracted using the E.Z.N.A. ® Mag-Bind Total Nucleic Acid Kit (Omega, USA), and the DNA samples were sent to Grandomics (Wuhan, China) for sequencing and assembly. The CrBV genome sequences have been uploaded to NCBI’s Genome database (GenBank accession numbers for 27 contigs: PQ863493–PQ863519).

### Transcriptome sequencing of FAW larvae

FAW larvae parasitized by *C. ruficru* were obtained using the same method. Whole larvae were collected at the 4th instar stage, and five larvae were placed in a 1.5 mL EP tube for total RNA extraction using Trizol reagent (Invitrogen, USA). RNA samples were subjected to quality control using an Agilent 2100 Bioanalyzer (Agilent, USA), ensuring accurate assessment of RNA integrity. The experiment was repeated three times. Following quality assessment, RNA samples were sent to Novogene Bioinformatics Technology Co. Ltd (Tianjin, China) for sequencing. All transcriptome files have been uploaded to NCBI’ s SRA database (Accession numbers: SRX23469543, SRX23469544, SRX23469545).

### Expression of CrBV genes in FAW larvae

Based on transcriptome analysis of parasitized FAW larvae, the top 20 CrBV genes with the highest expression in the larvae were selected. The expression of these genes was measured at 1, 3, 5, and 7 days post-parasitism, as well as in various tissues (head, body wall, fat body, hemolymph, midgut, salivary gland, and Malpighian tubule) at 5 days post-parasitism, using RT-qPCR. RNA extraction, cDNA synthesis, and RT-qPCR procedures followed the methods described in “RT-qPCR analysis of lipid metabolism genes in FAW larvae”. Each gene was analyzed with four biological replicates and three technical replicates.

### Lipid metabolism in FAW larvae following CrBV genes interference

Small interfering RNA (siRNA) targeting *CrBV3–31* (GCATGTTGTACGTGAAGCGCA), *CrBV11–6* (GCATCCTAGTGTTTGATAACG), and *CrBV13–7* (GGGACGAAGAAATGCTTTATA) were commercially designed and synthesized by Sangon Biotech (Shanghai) Co.,Ltd. (Shanghai, China). The siRNA targeting the green fluorescent protein (*GFP*) (GCCACAACGTCTATATCATGG, GenBank accession: MK078653.1) was used as a control. The synthesized siRNAs were dissolved in RNase-free water to achieve a final concentration of 2000 ng/µL. Parasitized FAW larvae were injected with 0.5 µL of siRNA on day 1 post-parasitism and 1 µL of siRNA on day 3 post-parasitism. At 48 hours following the second siRNA injection, whole FAW larvae were collected. Control larvae, parasitized and injected with si*GFP*, were also collected at the same time points. Silencing efficiency of *CrBV3–31*, *CrBV11–6*, and *CrBV13–7* were assessed by RT-qPCR as described in “RT-qPCR analysis of lipid metabolism genes in FAW larvae”, using six biological replicates and three technical replicates.

For the same experimental procedure, si*CrBV3–31*, *CrBV11–6*, and *CrBV13–7* were injected into parasitized FAW larvae, with si*GFP*-injected larvae serving as controls. At 48 hours post-injection, whole FAW larvae were collected to quantify the expression of seven genes (*FAS1*, *FAS2*, *GPAT1*, *GPAT4*, *DGAT1*, *LSD1*, *LSD2*) by RT-qPCR. Each gene was analyzed with six biological replicates and three technical replicates. Additionally, triglyceride concentrations in the fat bodies of FAW larvae were measured. Each treatment was repeated five times. Si*CrBV3–31* was injected into parasitized FAW larvae, with si*GFP*-injected larvae serving as controls. At 48 hours post-injection, lipid droplets were stained, and their sizes were measured by analyzing 200 lipid droplets per treatment.

For the same experimental procedure, si*CrBV3–31*, si*CrBV11–6*, and si*CrBV13–7* were injected into parasitized FAW larvae, with si*GFP*-injected larvae serving as controls. At 48 hours post-injection, whole FAW larvae were collected to quantify the expression of 19 other CrBV genes by RT-qPCR. Each gene was analyzed with six biological replicates and three technical replicates.

### Performance of parasitoid offspring following *CrBV3–31* interference

Parasitized FAW larvae were injected with 0.5 µL of si*CrBV3–31* on day 1 post-parasitism, 1 µL on day 3, 1 µL on day 5, and 1 µL on day 7 post-parasitism. Twenty-four hours after the fourth injection, whole FAW larvae were collected. Control groups included parasitized FAW larvae injected with si*GFP* and non-injected larvae. The silencing efficiency of si*CrBV3–31* was assessed by RT-qPCR as described in “RT-qPCR analysis of lipid metabolism genes in FAW larvae”. The experiment included five biological replicates and three technical replicates.

Following the same procedure, si*CrBV3–31* was injected into parasitized FAW larvae four times consecutively, with si*GFP*-injected and non-injected larvae serving as controls. The parasitoid larvae were reared until emergence from the host larvae, followed by pupation. The number of parasitoid cocoons produced by each host was recorded, with data collected from 45 FAW larvae per treatment. For each treatment, 10 one-day-old parasitoid cocoons were collected and weighed, and the experiment was repeated 10 times. Additionally, one-day-old cocoons were dissected to extract the pupae for triglyceride concentration analysis, with each treatment repeated five times.

The parasitoid cocoons were further reared until adult emergence, at which point developmental time and eclosion rates were recorded. Data were collected from 45 FAW larvae per treatment. The number of eggs in the ovaries of female parasitoids (emerged within 12 hours) was also recorded, with each treatment repeated 30 times. The hind tibia lengths of female and male parasitoid adults were measured using a microscope (JT-H3, Shenzhen Jingtou Youcheng, China), with each treatment repeated 30 times.

To evaluate the parasitizing efficacy of *C. ruficrus* offspring using FAW larvae as hosts after the *CrBV3–31* gene interference in the parasitized FAW larvae, newly emerged females and males (emerged within 12 hours) were paired for 1 day for mating. The mated females were then placed in a rearing container (8.5 cm diameter, 6.0 cm height, with a gauze mesh in the center of the lid) containing 10 third instar FAW larvae (one-day-old). Cotton balls soaked in 10% honey water were provided as supplemental nutrition. After 8 hours, FAW larvae were collected and dissected to check for parasitoid eggs, and the number of parasitized larvae was recorded. Each treatment was repeated 10 times. Additionally, parasitoid cocoons that failed to emerge were dissected to determine their developmental status, with 30 cocoons examined per treatment and repeated five times.

### Construction of a phylogenetic tree

The amino acid sequence of one *LCT* gene from CrBV was submitted to the NCBI database for a BlastP alignment. Subsequently, the amino acid sequences of *LCT* genes from bracoviruses with higher alignment scores were downloaded. Additionally, the *LCT* gene sequence of a more distantly related FAW species was retrieved as an outgroup. All *LCT* gene sequences were imported into MEGA11.0 software, and multiple sequence alignment was performed using ClustalW. A phylogenetic tree was then constructed using the Maximum Likelihood (ML) method based on the multiple sequence alignment, with branch support evaluated through 1000 bootstrap iterations. The same approach was used to construct phylogenetic trees for the three *ANK* genes from CrBV in comparison to *ANK* genes from other species, as well as for the *LSD1* gene of FAW and *LSD1* genes from other species.

### Statistical analyses

Normality (Shapiro-Wilk test) and homogeneity of variance (Levene’s test) were first assessed for all datasets. For data meeting the assumptions of normality and homogeneity of variance, one-way ANOVA was performed, followed by mean separation using the Duncan test at *P* < 0.05. Student’s t-test was used for pairwise comparisons. For data that did not meet these assumptions, the Kruskal-Wallis H-test was used for multiple treatment comparisons, and the Mann-Whitney U-test was employed for pairwise comparisons. The data were denoted as means ± standard error (SE) and analyzed using the statistical software package SPSS 23.0. Graphs were generated using GraphPad Prism 8 software.

## Supporting information

S1 Fig Genomic analysis of CrBV. A. Morphology of CrBV particles. B. Circular representation of the CrBV genome. C. Functional annotation of the CrBV genome. ★ indicates incomplete sequence.
(TIF)

S2 Fig Phylogenetic relationship of LCT in CrBV and other species. The LCT of *Cotesia ruficrus* bracovirus is highlighted in red.
(TIF)

S3 Fig Phylogenetic relationship of ANK in CrBV and other species. The ANK of *Cotesia ruficrus* bracovirus is highlighted in red.
(TIF)

S4 Fig Venn diagram of co-expression of CrBV genes (A) and cluster heatmaps of gene expression levels in parasitized FAW (B). In the heatmap, red indicates higher gene expression level, while blue represents lower expression levels.
(TIF)

S5 Fig Phylogenetic relationship of LSD1 in *Spodoptera frugiperda* and other species. The LSD1 of *Spodoptera frugiperda* is denoted by asterisks (★). *Cotesia glomerata* (Cglo), *Microplitis demolitor* (Mdem), *Microplitis mediator* (Mmed), *Frankliniella fusca* (Ffus), *Frankliniella occidentalis* (Focc), *Nilaparvata lugens* (Nlug), *Planococcus citri* (Pcit), *Acyrthosiphon pisum* (Apis), *Aphis gossypii* (Agos), *Zootermopsis nevadensis* (Znev), *Cryptotermes secundus* (Csec), *Gryllus bimaculatus* (Gbim), *Schistocerca americana* (Same), *Schistocerca cancellata* (Scan), *Schistocerca gregaria* (Sgre), *Coccinella septempunctata* (Csep), *Agrilus planipennis* (Apla), *Cylas formicarius* (Cfor), *Anthonomus grandis* (Agra), *Dendroctonus ponderosae* (Dpon), *Tribolium castaneum* (Tcas), *Anoplophora glabripennis* (Agla), *Aethina tumida* (Atum), *Sabethes cyaneus* (Scya), *Aedes aegypti* (Aaeg), *Drosophila mojavensis* (Dmoj), *Drosophila montana* (Dmon), *Drosophila virilis* (Dvir), *Drosophila suzukii* (Dsuz), *Drosophila teissieri* (Dtei), *Drosophila simulans* (Dsim), *Drosophila melanogaster* (Dmel), *Plutella xylostella* (Pxyl), *Cydia splendana* (Cspl), *Plodia interpunctella* (Pint), *Amyelois transitella* (Atra), *Bombyx mori* (Bmor), *Manduca sexta* (Msex), *Hyposmocoma kahamanoa* (Hkah), *Pectinophora gossypiella* (Pgos), *Trichoplusia ni* (Tni), *Helicoverpa zea* (Hzea), *Spodoptera litura* (Slit), *Spodoptera frugiperda* (Sfru), *Aricia agestis* (Aage), *Zerene cesonia* (Zces), *Colias croceus* (Ccro), *Pieris napi* (Pnap), *Pieris brassicae* (Pbra), *Pieris rapae* (Prap), *Maniola hyperantus* (Mhyp), *Maniola jurtina* (Mjur), *Maniola cinxia* (Mcin), *Nymphalis io* (Nio), *Vanessa tameamea* (Vtam), *Vanessa cardui* (Vcar), *Vanessa atalanta* (Vata).
(TIF)

S6 Fig Expression of 20 CrBV genes in FAW following *CrBV3–31* interference. Data were analyzed using Student’s t-test. Values are presented as means ± SE. * indicates significant differences between si*GFP*-injected and si*CrBV3–31*-injected FAW larvae at *P *< 0.05.
(TIF)

S7 Fig Expression of 20 CrBV genes in FAW following *CrBV11–6* interference. Data were analyzed using Student’s t-test. Values are presented as means ± SE. *, ** indicate significant differences between si*GFP*-injected and si*CrBV11–6*-injected FAW larvae at *P *< 0.05 and *P *< 0.01, respectively.
(TIF)

S8 Fig Expression of 20 CrBV genes in FAW following *CrBV13–7* interference. Data were analyzed using Student’s t-test. Values are presented as means ± SE. *, ** indicate significant differences between si*GFP*-injected and si*CrBV13–7*-injected FAW larvae at *P *< 0.05 and *P *< 0.01, respectively.
(TIF)

S9 Fig Developmental status of non-eclosing *Cotesia ruficrus.* A. Four developmental stages of non-eclosing *C. ruficrus*: (a) white pupae, (b) black pupae, (c) malformed adults, (d) normal but deceased adults. B. Number of non-eclosing *C. ruficrus* in different developmental stages. P, P + si*GFP*, and P + si*CrBV3–31* represent non-injected, si*GFP*-injected, and si*CrBV3–31*-injected parasitized FAW larvae, respectively. Data were analyzed using one-way ANOVA (Duncan’s test). Values are expressed as means ± SE.
(TIF)

S1 Table Assembly result statistics of CrBV genome. (DOCX)

S2 Table Contig list of the CrBV genome. (DOCX)

S3 Table Genomic features of CrBV. (DOCX)

S4 Table Functional annotation statistics of coding proteins in the CrBV Genome. (DOCX)

S5 Table Comparative analysis of 11 bracovirus genomes. (DOCX)

S6 Table Information of ten genes involved in lipid metabolism. (DOCX)

S7 Table List of primer sequences. (DOCX)
