# Supplementary figures and images for: Regulation of lipid metabolism in Spodoptera frugiperda by the symbiotic bracovirus of the gregarious parasitoid Cotesia ruficrus
Source: PLoS Pathog. 2025 Oct 17;21(10):e1013605. doi: 10.1371/journal.ppat.1013605 (PMC12548909; doi:10.1371/journal.ppat.1013605)

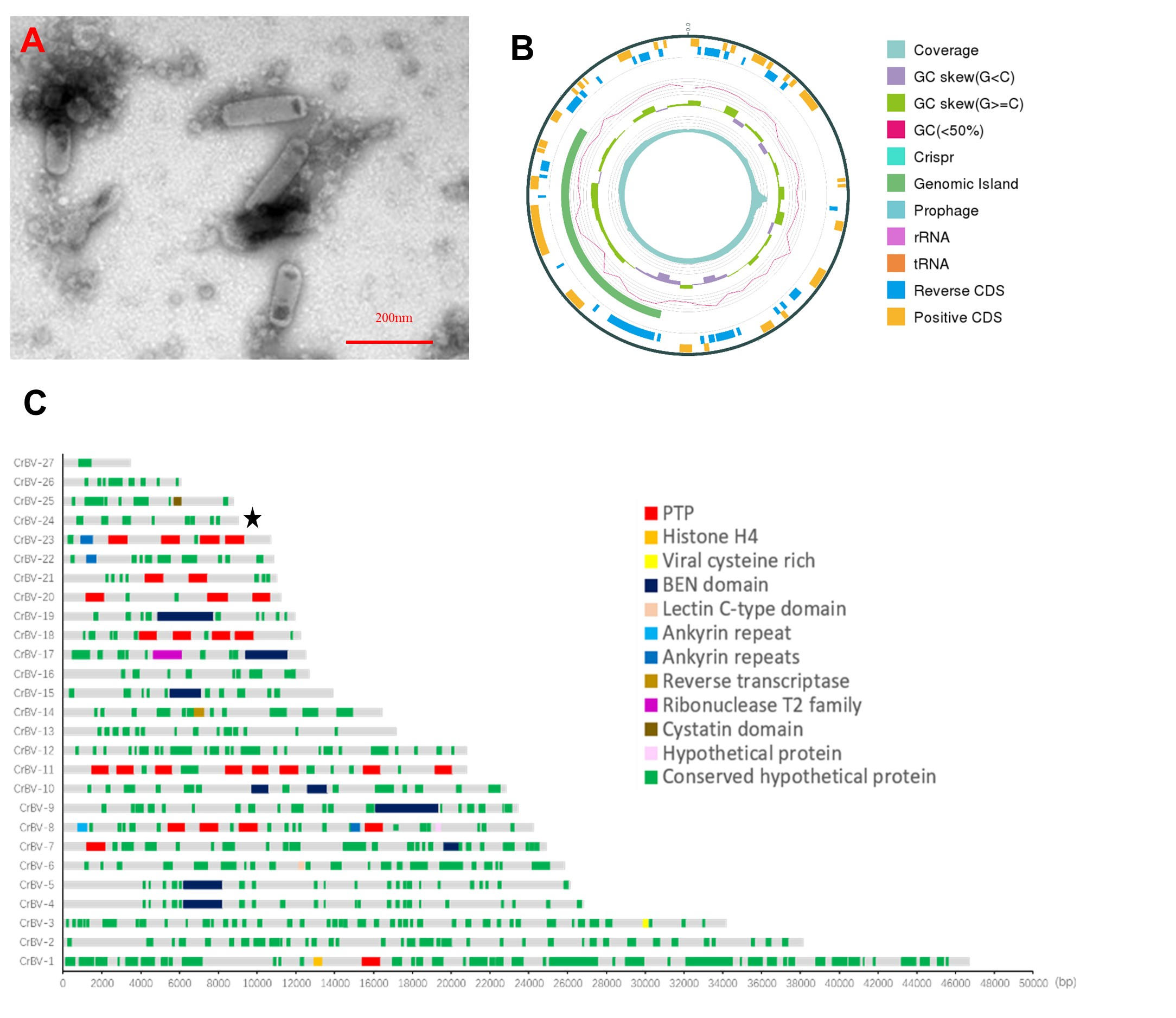

Supplement: S1 Fig — A. Morphology of CrBV particles. B. Circular representation of the CrBV genome. C. Functional annotation of the CrBV genome. ★ indicates incomplete sequence. (TIF) [file ppat.1013605.s001.tif]

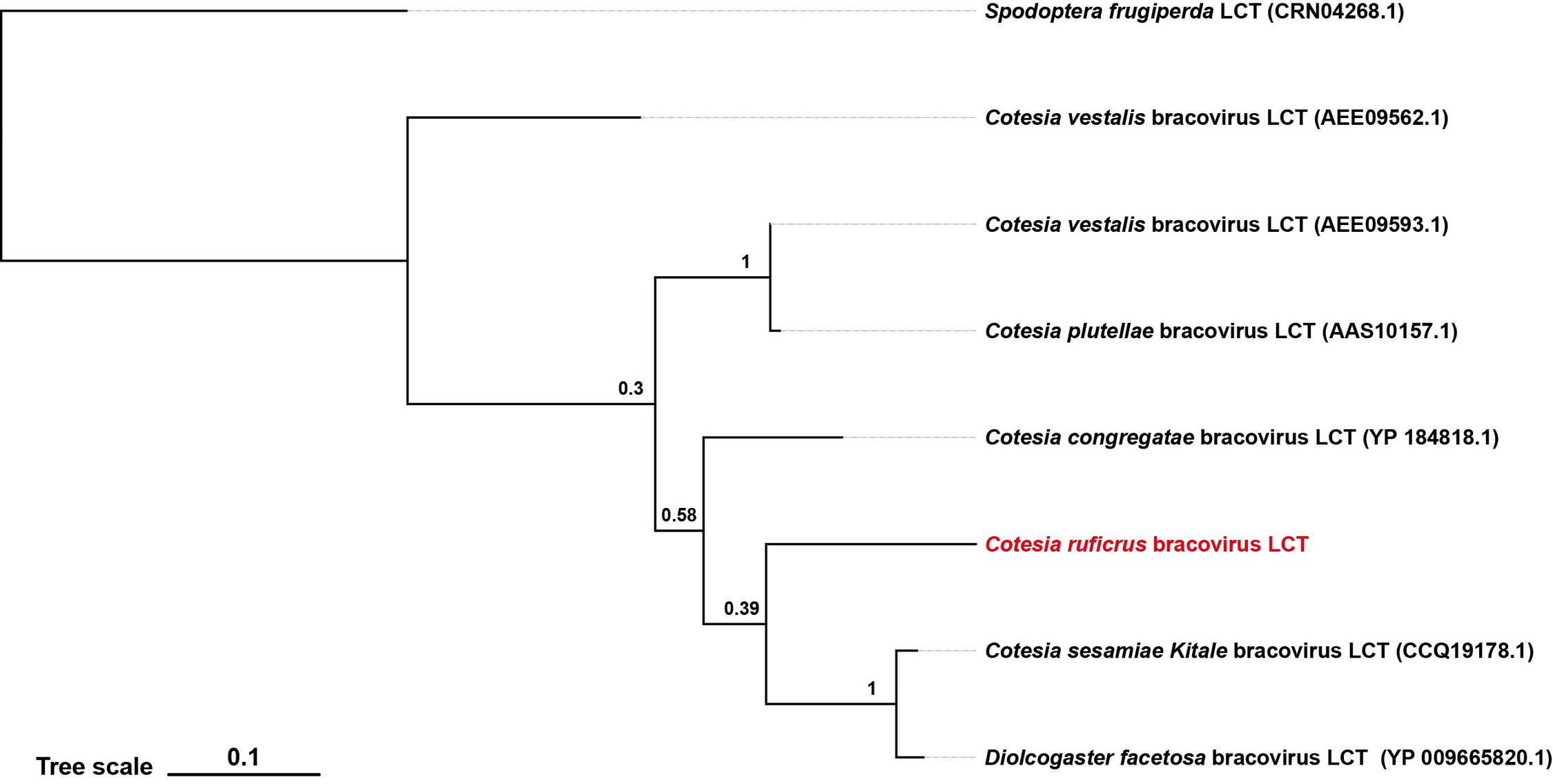

Supplement: S2 Fig — The LCT of Cotesia ruficrus bracovirus is highlighted in red. (TIF) [file ppat.1013605.s002.tif]

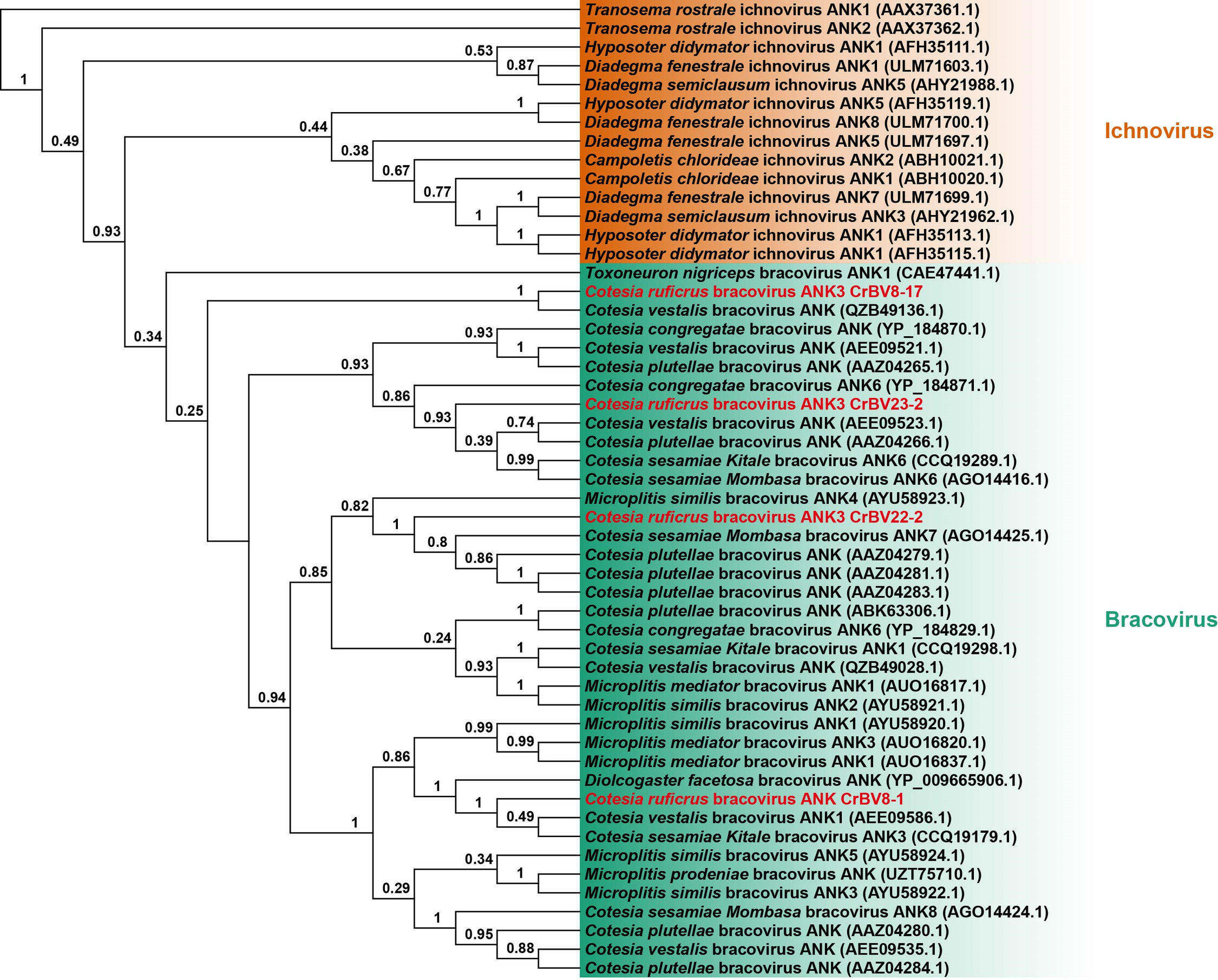

Supplement: S3 Fig — The ANK of Cotesia ruficrus bracovirus is highlighted in red. (TIF) [file ppat.1013605.s003.tif]

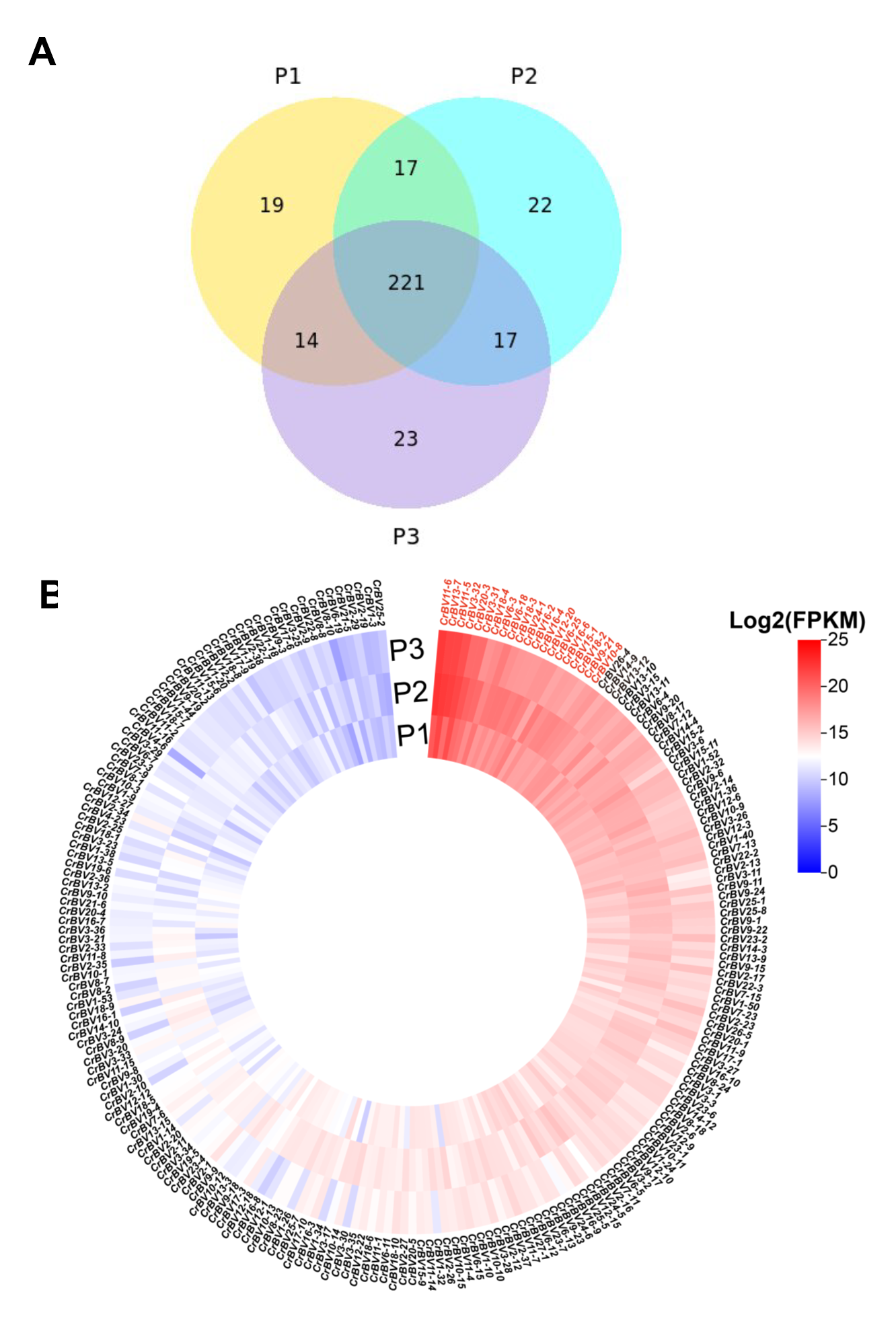

Supplement: S4 Fig — In the heatmap, red indicates higher gene expression level, while blue represents lower expression levels. (TIF) [file ppat.1013605.s004.tif]

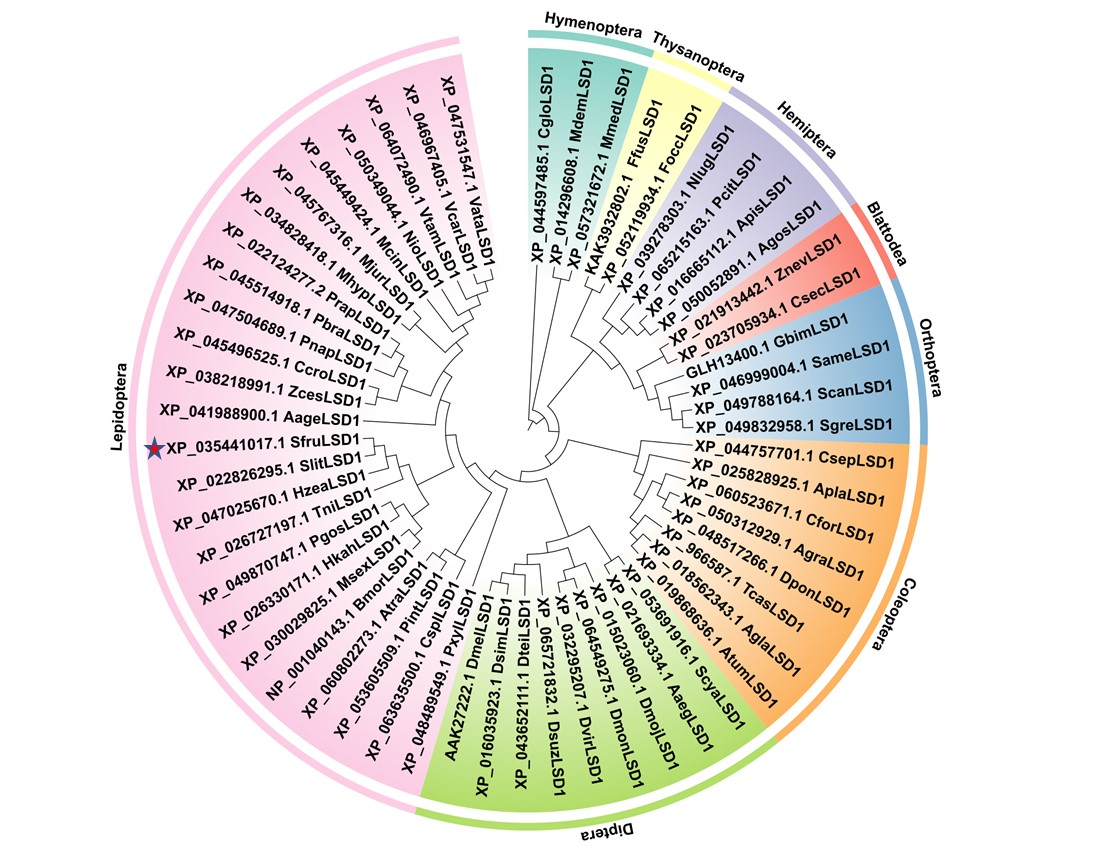

Supplement: S5 Fig — The LSD1 of Spodoptera frugiperda is denoted by asterisks (★). Cotesia glomerata (Cglo), Microplitis demolitor (Mdem), Microplitis mediator (Mmed), Frankliniella fusca (Ffus), Frankliniella occidentalis (Focc), Nilaparvata lugens (Nlug), Planococcus citri (Pcit), Acyrthosiphon pisum (Apis), Aphis gossypii (Agos), Zootermopsis nevadensis (Znev), Cryptotermes secundus (Csec), Gryllus bimaculatus (Gbim), Schistocerca americana (Same), Schistocerca cancellata (Scan), Schistocerca gregaria (Sgre), Coccinella septempunctata (Csep), Agrilus planipennis (Apla), Cylas formicarius (Cfor), Anthonomus grandis (Agra), Dendroctonus ponderosae (Dpon), Tribolium castaneum (Tcas), Anoplophora glabripennis (Agla), Aethina tumida (Atum), Sabethes cyaneus (Scya), Aedes aegypti (Aaeg), Drosophila mojavensis (Dmoj), Drosophila montana (Dmon), Drosophila virilis (Dvir), Drosophila suzukii (Dsuz), Drosophila teissieri (Dtei), Drosophila simulans (Dsim), Drosophila melanogaster (Dmel), Plutella xylostella (Pxyl), Cydia splendana (Cspl), Plodia interpunctella (Pint), Amyelois transitella (Atra), Bombyx mori (Bmor), Manduca sexta (Msex), Hyposmocoma kahamanoa (Hkah), Pectinophora gossypiella (Pgos), Trichoplusia ni (Tni), Helicoverpa zea (Hzea), Spodoptera litura (Slit), Spodoptera frugiperda (Sfru), Aricia agestis (Aage), Zerene cesonia (Zces), Colias croceus (Ccro), Pieris napi (Pnap), Pieris brassicae (Pbra), Pieris rapae (Prap), Maniola hyperantus (Mhyp), Maniola jurtina (Mjur), Maniola cinxia (Mcin), Nymphalis io (Nio), Vanessa tameamea (Vtam), Vanessa cardui (Vcar), Vanessa atalanta (Vata). (TIF) [file ppat.1013605.s005.tif]

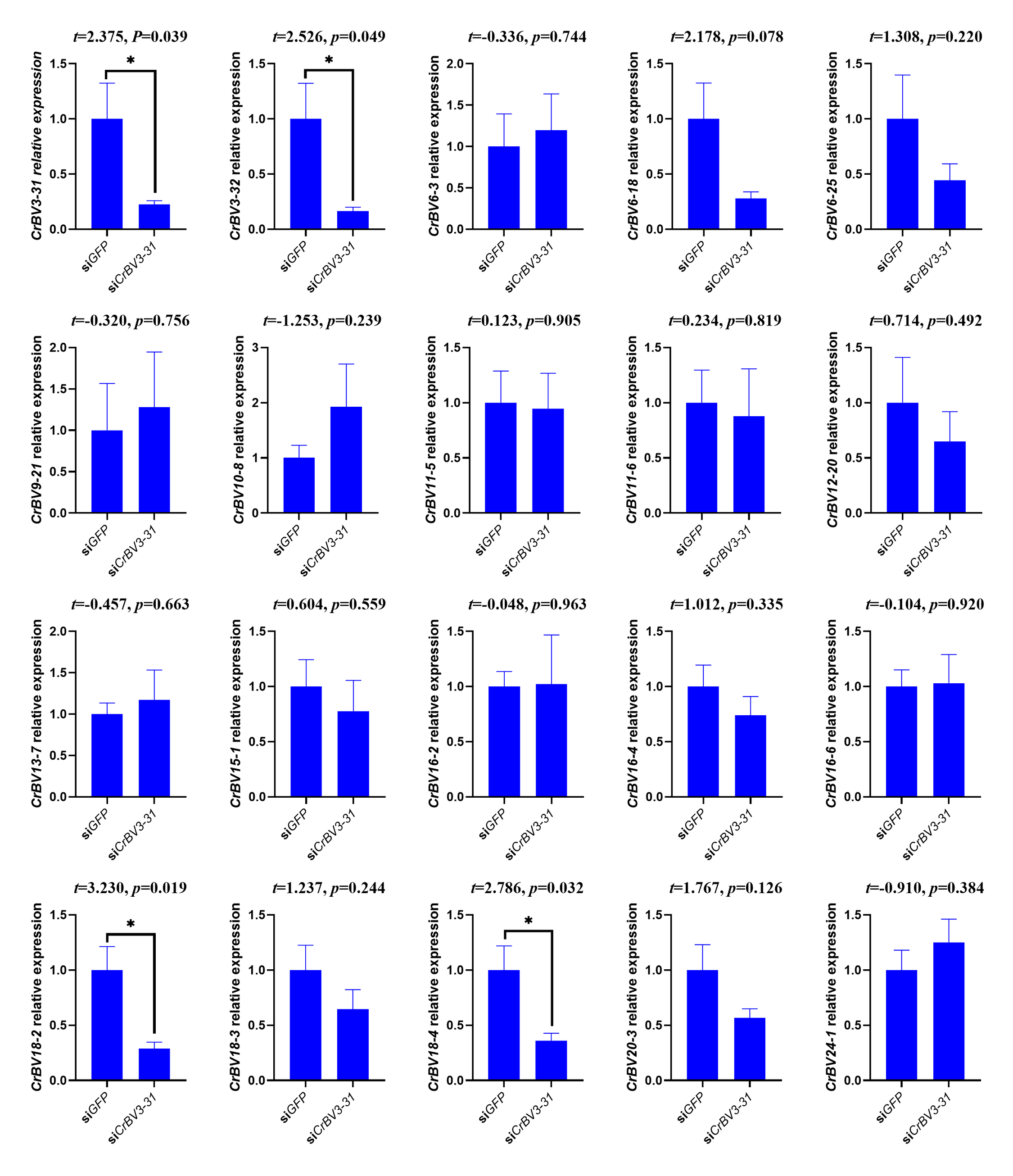

Supplement: S6 Fig — Data were analyzed using Student’s t-test. Values are presented as means ± SE. * indicates significant differences between siGFP-injected and siCrBV3–31-injected FAW larvae at P < 0.05. (TIF) [file ppat.1013605.s006.tif]

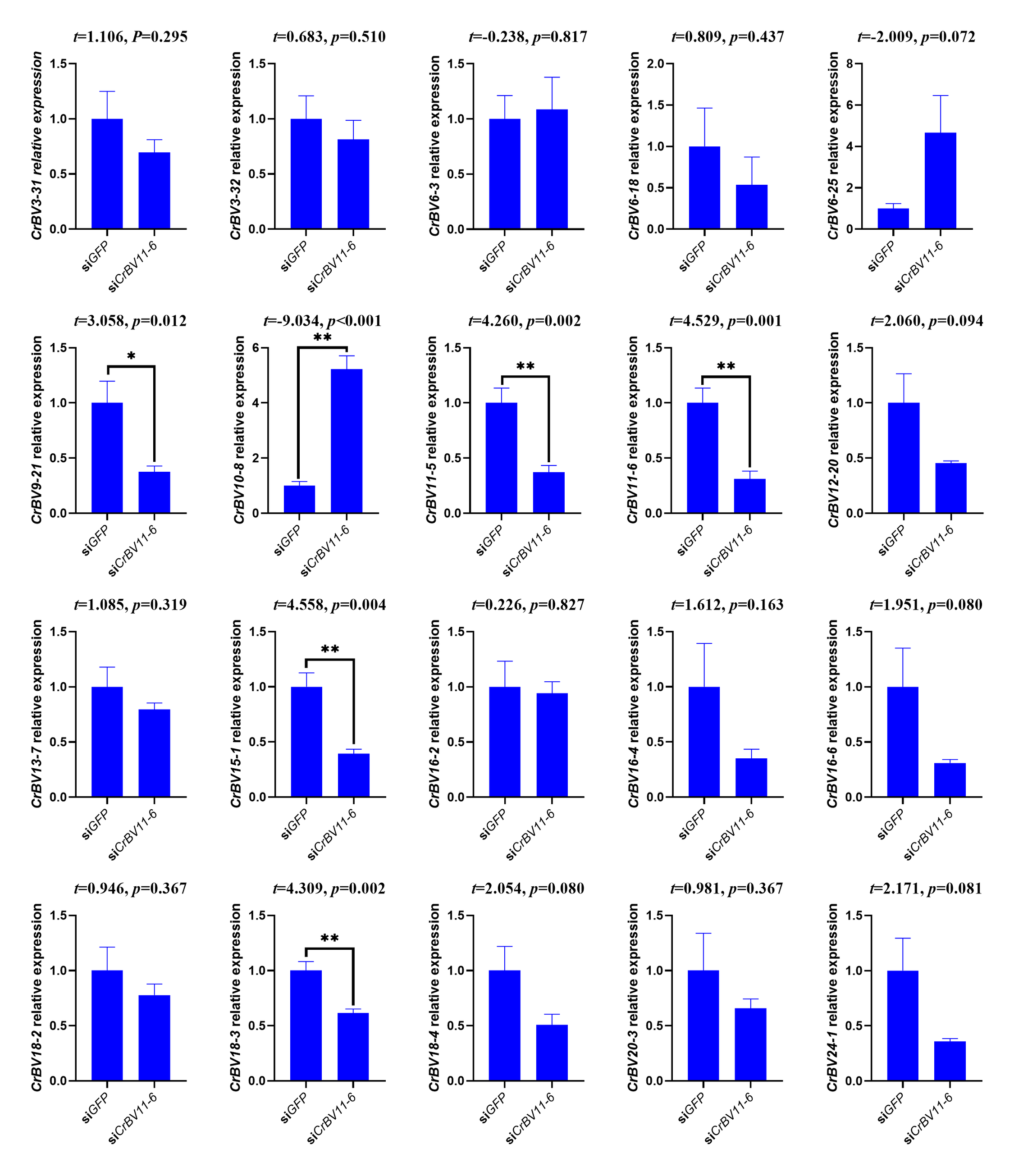

Supplement: S7 Fig — Data were analyzed using Student’s t-test. Values are presented as means ± SE. *, ** indicate significant differences between siGFP-injected and siCrBV11–6-injected FAW larvae at P < 0.05 and P < 0.01, respectively. (TIF) [file ppat.1013605.s007.tif]

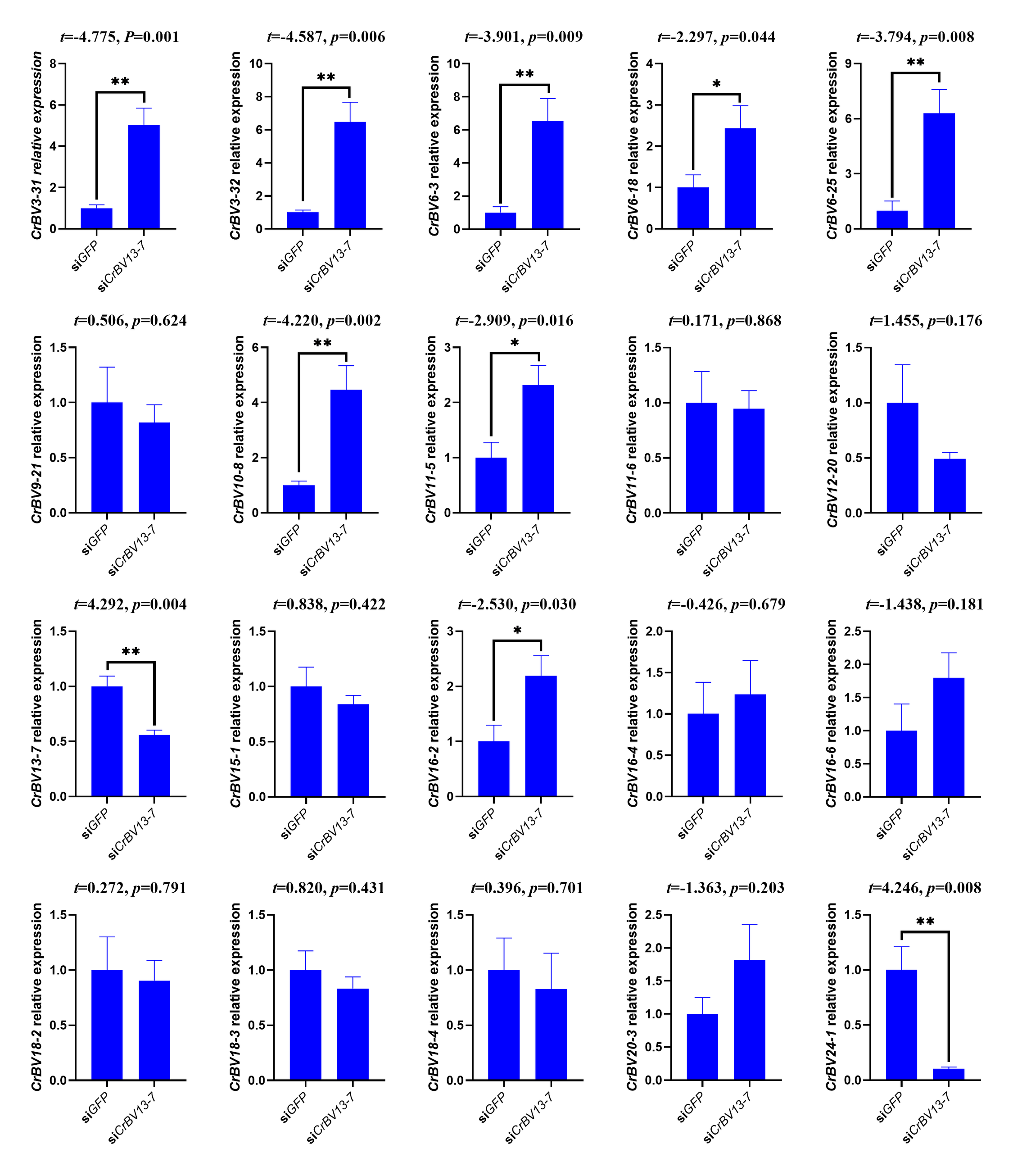

Supplement: S8 Fig — Data were analyzed using Student’s t-test. Values are presented as means ± SE. *, ** indicate significant differences between siGFP-injected and siCrBV13–7-injected FAW larvae at P < 0.05 and P < 0.01, respectively. (TIF) [file ppat.1013605.s008.tif]

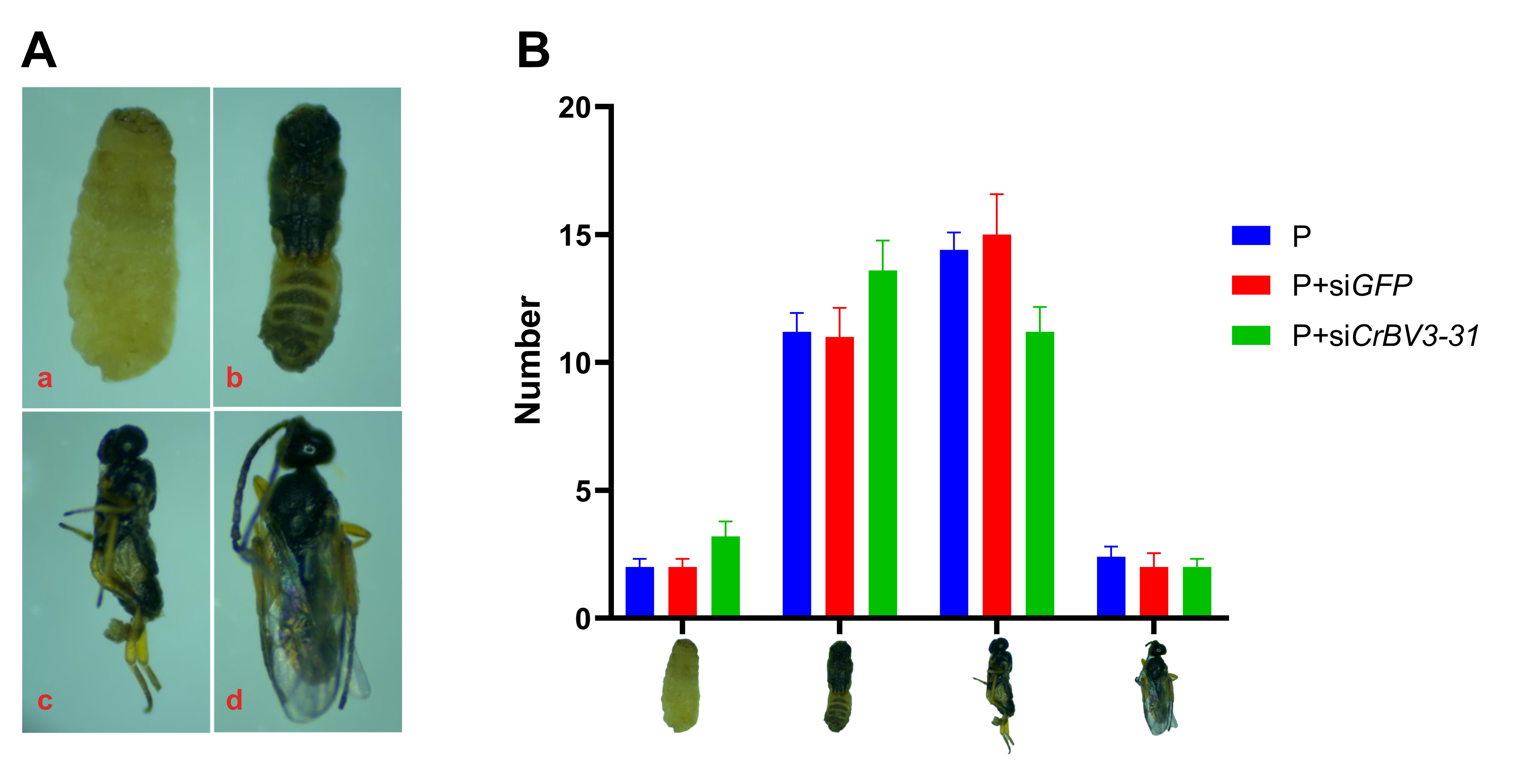

Supplement: S9 Fig — A. Four developmental stages of non-eclosing C. ruficrus: (a) white pupae, (b) black pupae, (c) malformed adults, (d) normal but deceased adults. B. Number of non-eclosing C. ruficrus in different developmental stages. P, P + siGFP, and P + siCrBV3–31 represent non-injected, siGFP-injected, and siCrBV3–31-injected parasitized FAW larvae, respectively. Data were analyzed using one-way ANOVA (Duncan’s test). Values are expressed as means ± SE. (TIF) [file ppat.1013605.s009.tif]
