# Supplementary material for: Regulation of lipid metabolism in Spodoptera frugiperda by the symbiotic bracovirus of the gregarious parasitoid Cotesia ruficrus
Source: PLoS Pathog. 2025 Oct 17;21(10):e1013605. doi: 10.1371/journal.ppat.1013605 (PMC12548909; doi:10.1371/journal.ppat.1013605)
Supplement: S1 Table — (DOCX) [file ppat.1013605.s010.docx]

**S1_Table.** **Assembly result statistics of CrBV genome**

| Genome size (bp) | Contig Number | Contig N50 (bp) | Longest contig (bp) | Shortest contig (bp) |
| --- | --- | --- | --- | --- |
| 503,647 | 27 | 23,455 | 46,703 | 3,473 |
