# Supplementary material for: Regulation of lipid metabolism in Spodoptera frugiperda by the symbiotic bracovirus of the gregarious parasitoid Cotesia ruficrus
Source: PLoS Pathog. 2025 Oct 17;21(10):e1013605. doi: 10.1371/journal.ppat.1013605 (PMC12548909; doi:10.1371/journal.ppat.1013605)
Supplement: S2 Table — (DOCX) [file ppat.1013605.s011.docx]

**S2_Table.** **Contig list of the CrBV genome**

| Contig | Length (bp) | GC content (%) | Topology | Sequencing depth (X) |
| --- | --- | --- | --- | --- |
| CrBV-1 | 46,703 | 34.32 | circular | 2645.25 |
| CrBV-2 | 38,129 | 34.66 | circular | 1453.09 |
| CrBV-3 | 34,165 | 35.10 | circular | 1542.46 |
| CrBV-4 | 26,821 | 34.57 | circular | 926.82 |
| CrBV-5 | 26,138 | 34.75 | circular | 557.63 |
| CrBV-6 | 25,846 | 34.04 | circular | 3457.21 |
| CrBV-7 | 24,894 | 33.94 | circular | 1985.03 |
| CrBV-8 | 24,240 | 35.42 | circular | 1128.87 |
| CrBV-9 | 23,455 | 35.83 | circular | 1430.86 |
| CrBV-10 | 22,835 | 35.44 | circular | 1468.67 |
| CrBV-11 | 21,619 | 34.73 | circular | 4888.84 |
| CrBV-12 | 20,791 | 33.96 | circular | 3361.07 |
| CrBV-13 | 17,159 | 32.97 | circular | 1555.78 |
| CrBV-14 | 16,430 | 33.58 | circular | 3525.26 |
| CrBV-15 | 13,897 | 34.14 | circular | 3470.36 |
| CrBV-16 | 12,695 | 33.13 | circular | 1029.19 |
| CrBV-17 | 12,501 | 35.76 | circular | 2185.12 |
| CrBV-18 | 12,228 | 35.26 | circular | 1895.80 |
| CrBV-19 | 11,951 | 34.15 | circular | 1804.77 |
| CrBV-20 | 11,230 | 34.74 | circular | 1802.14 |
| CrBV-21 | 11,014 | 31.99 | circular | 700.99 |
| CrBV-22 | 10,851 | 37.96 | circular | 1219.59 |
| CrBV-23 | 10,684 | 35.16 | circular | 2369.72 |
| CrBV-24 | 9,041 | 35.43 | circular | 737.32 |
| CrBV-25 | 8,788 | 35.32 | linear (incomplete) | 2167.08 |
| CrBV-26 | 6,069 | 32.56 | circular | 2297.93 |
| CrBV-27 | 3,473 | 30.81 | circular | 661.69 |
