# Supplementary material for: Regulation of lipid metabolism in Spodoptera frugiperda by the symbiotic bracovirus of the gregarious parasitoid Cotesia ruficrus
Source: PLoS Pathog. 2025 Oct 17;21(10):e1013605. doi: 10.1371/journal.ppat.1013605 (PMC12548909; doi:10.1371/journal.ppat.1013605)
Supplement: S3 Table — (DOCX) [file ppat.1013605.s012.docx]

**S3_Table.** **Genomic features of CrBV**

| Feature | CrBV |
| --- | --- |
| Genome size | 503,647 bp |
| Number of circles | 27 |
| G+C content | 33.41% |
| Predicted genes | 483 |
| Coding density | 34.63% |
| Predicted tRNAs | 5 |
| Genomic island | 25 |
