# Supplementary material for: Regulation of lipid metabolism in Spodoptera frugiperda by the symbiotic bracovirus of the gregarious parasitoid Cotesia ruficrus
Source: PLoS Pathog. 2025 Oct 17;21(10):e1013605. doi: 10.1371/journal.ppat.1013605 (PMC12548909; doi:10.1371/journal.ppat.1013605)
Supplement: S4 Table — (DOCX) [file ppat.1013605.s013.docx]

**S4_Table.** **Functional annotation statistics of coding proteins in the CrBV Genome**

| Family | number |
| --- | --- |
| PTP | 27 |
| Histone H4 | 1 |
| Viral cysteine rich | 1 |
| BEN domain | 9 |
| Lectin C-type domain | 1 |
| Ankyrin repeat | 1 |
| Ankyrin repeats (3 copies) | 3 |
| Reverse transcriptase (RNA-dependent DNA polymerase) | 1 |
| Ribonuclease T2 family | 1 |
| Cystatin domain | 1 |
| Hypothetical protein | 1 |
