# Supplementary material for: Regulation of lipid metabolism in Spodoptera frugiperda by the symbiotic bracovirus of the gregarious parasitoid Cotesia ruficrus
Source: PLoS Pathog. 2025 Oct 17;21(10):e1013605. doi: 10.1371/journal.ppat.1013605 (PMC12548909; doi:10.1371/journal.ppat.1013605)
Supplement: S5 Table — (DOCX) [file ppat.1013605.s014.docx]

**S5_Table.** **Comparative analysis of 12 bracovirus genomes**

| Bracovirus species | Genome size | Number of circles | G+C content | Predicted genes | Coding density | References |
| --- | --- | --- | --- | --- | --- | --- |
| *Cotesia ruficrus* bracovirus | 503.647 kb | 27 | 33.41% | 483 | 34.63% |  |
| *Cotesia congregata* bracovirus | 567.7 kb | 30 | 33.5% | 260 | 22% | Espagne et al. 2004 |
| *Cotesia vestalis* bracovirus | 540.2 kb | 35 | 34.5% | 157 | 27% | Chen et al. 2011 |
| *Cotesia sesamiae Kitale* bracovirus | 373 kb | 26 | 34.1% | 139 | 27% | Jancek et al. 2013 |
| *Cotesia sesamiae Mombasa* bracovirus | 231 kb | 16 | 39% | 88 | 24% | Jancek et al. 2013 |
| *Cotesia plutellae* bracovirus | 351.299 kb | 24 | 34.6% | 125 | 32.3% | Choi et al. 2009 |
| *Chelonus inanitus* bracovirus | 161.7 kb | 18 | 32% | 30 |  | Wyder et al. 2002 |
| *Microplitis demolitor* bracovirus | 185.2 kb | 15 | 34% | 61 | 17% | Webb et al. 2006 |
| *Glyptapanteles indiensis* bracovirus | 508.6 kb | 27 | 36% | 197 | 33% | Desjardins et al. 2008 |
| *Glyptapanteles flavicoxis* bracovirus | 594 kb | 29 | 35% | 193 | 32% | Desjardins et al. 2008 |
| *Toxoneuron nigriceps Bracovirus* | 203.236 kb | 27 | 32.6% | 42 | 22% | Ibrahim 2010 |
| *Diolcogaster facetosa* bracovirus | 787.3 kb | 29 | 35% | 112 |  | ASM283394v2, Jun 28, 2019 |
