# Supplementary material for: Regulation of lipid metabolism in Spodoptera frugiperda by the symbiotic bracovirus of the gregarious parasitoid Cotesia ruficrus
Source: PLoS Pathog. 2025 Oct 17;21(10):e1013605. doi: 10.1371/journal.ppat.1013605 (PMC12548909; doi:10.1371/journal.ppat.1013605)
Supplement: S6 Table — (DOCX) [file ppat.1013605.s015.docx]

**S6_Table.** **Information of ten genes involved in lipid metabolism**

| Gene name | Gene ID | Gene chr | Gene length | Gene description |
| --- | --- | --- | --- | --- |
| FAS1 | 118274609 | NC_049729.1 | 7875 | fatty acid synthase |
| FAS2 | 118274634 | NC_049729.1 | 7857 | fatty acid synthase |
| GPAT1 | 118270204 | NC_049723.1 | 2260 | glycerol-3-phosphate acyltransferase 1 |
| GPAT4 | 118278786 | NC_049735.1 | 1932 | glycerol-3-phosphate acyltransferase 4 |
| AGPAT3 | 118262091 | NC_049712.1 | 4168 | 1-Acylglycerol-3-phosphate O-acyltransferase 1 |
| LPIN | 118271232 | NC_049711.1 | 4089 | phosphatidate phosphatase LPIN |
| PLD1 | 118263587 | NC_049714.1 | 4433 | Phospholipase D 1 |
| DGAT1 | 118273106 | NC_049727.1 | 2584 | diacylglycerol O-acyltransferase |
| LSD1 | 118269804 | NC_049722.1 | 1682 | lipid storage droplet-1 |
| LSD2 | 118269580 | NC_049722.1 | 1561 | lipid storage droplet-2 |
