# Supplementary material for: Regulation of lipid metabolism in Spodoptera frugiperda by the symbiotic bracovirus of the gregarious parasitoid Cotesia ruficrus
Source: PLoS Pathog. 2025 Oct 17;21(10):e1013605. doi: 10.1371/journal.ppat.1013605 (PMC12548909; doi:10.1371/journal.ppat.1013605)
Supplement: S7 Table — (DOCX) [file ppat.1013605.s016.docx]

**S7_Table.** **List of primer sequences**

| Gene name | Forward | Reverse |
| --- | --- | --- |
| FAS1 | CGTGTCCTGTGGTCGTGGTAAC | TCCTCCATTCGCAGATCCTCTCC |
| FAS2 | TGAGTCTGGATGTGAGGAGATGCT | TGGTGCGAACGATGTCTTGAAGG |
| GPAT1 | TCCTACACCGTCTCCGACAAGC | ATGGCGACACGTCACCGAGTA |
| GPAT4 | ATCCTCGGTCCTTCGCCAGAAT | TGTGTGGTCTTGGGTCCGTTCT |
| AGPAT3 | TTGTACGGCAAGCCGGTCAAC | CGCTGCTTCACCTTCGTCTTCA |
| LIPIN | CCAGGTGGTGCAGAAGATCAGC | CTTTCCGTTTCCGCCGTTAGGT |
| PLD1 | GGTCTGCTGTGTGCCAAATG | CCCGTTGAGTACATTCCGCT |
| DGAT1 | GCTGCTACAATTCGCTGATCGC | TCCAAGTGCTCCAGAACACGGA |
| LSD1 | CCGACCTGCTACACTGGAGGAA | ATGCCTTGACGGACGTTCTTGG |
| LSD2 | TGCCACTCGTCACAGAACAACC | TCGCCCATGACAGCTCCTTGA |
| CrBV3-31 | CGCAGAGACAGCACGCCTTT | CGTCCTAGCCCTTCCACGTACT |
| CrBV3-32 | ACTCCCTACCCATCAGCAGACC | ACCCTGGCACTCGTTCCATTCT |
| CrBV6-3 | GCGAGGCATTTAAACTGGGACC | TGGAAGTTCCATGAGGGTTGCT |
| CrBV6-18 | CCTGGCTACGAATCCAGCGT | TTCCTTGAGCAGGTTCAACGCA |
| CrBV6-25 | GATCGCAGCGGGGTATCTG | GAGCTTGCTTCTGTGGGGTA |
| CrBV9-21 | GCTGAAGACGTTTGCCAACCAC | TGCCATCACATCATTCGCCGAT |
| CrBV10-8 | TGCAAGTCATTAGCGCGGATGT | GGCTCTTGCGCCAGTAAACGA |
| CrBV11-5 | ACAACAACCTGCTCGCTCACG | TTGTACCGACATGTGCCTGTGC |
| CrBV11-6 | AATCGATTCACCGGACCAGG | GCAGTGACTTCATATCCCGCT |
| CrBV12-20 | AGGGAGCAACTCGCATGGTACA | TGTCCACTTGCTGGAGGAGACC |
| CrBV13-7 | GCTGCTTATGATGCGGCCCATT | CCCGGCATCAGTGAAGAACAGG |
| CrBV15-1 | ATTGCGATGGCGGGTGCATC | GTTGCGTGTCTCTGCTCCGTAG |
| CrBV16-2 | ATGGCACCACTGCTAAGATCATCA | TTTATTCTTCTTCGCGGGACCTTT |
| CrBV16-4 | GTGCTTCTCGCTGTAGTGCT | GGCACAAAAACTGTGTTGGACA |
| CrBV16-6 | ATCGGGTTCAAAGGAGGATGCA | AGCCATCTTTCACGAGGTGGTT |
| CrBV18-2 | CAGGCAATTCCGGTGCAGCT | CATGGTTAAGATCGCGCACAGA |
| CrBV18-3 | TGCCTCTAATTTTTGGACTTGAACA | CCACCACAATTTTGGACGGT |
| CrBV18-4 | CGGTGGAGATTGCACGACAGTT | TGATAACTAGCGCCGACACGTT |
| CrBV20-3 | GGCCATTGAAGGCTACCGGAAA | CAAAGCCGACACGCCGTCAA |
| CrBV24-1 | GCTGTGGACCGACTTGTGCTT | CGAACTGTACTGGCGTGGGTTT |
| LOC118272864 (reference gene) | CGTATCAACCGACCTCCACT | AGGCACCTTGTAGAGCCTCA |
